# Single-cell transcriptomics of popliteal lymphatic vessels and peripheral veins reveals altered lymphatic muscle and immune cell populations in the TNF-Tg arthritis model

**DOI:** 10.1186/s13075-022-02730-z

**Published:** 2022-03-07

**Authors:** H. Mark Kenney, Chia-Lung Wu, Alayna E. Loiselle, Lianping Xing, Christopher T. Ritchlin, Edward M. Schwarz

**Affiliations:** 1 Center for Musculoskeletal Research, University of Rochester Medical Center, 601 Elmwood Ave, Box 665, Rochester, NY 14642 USA; 2 Department of Pathology & Laboratory Medicine, University of Rochester Medical Center, Rochester, NY USA; 3 Department of Orthopaedics, University of Rochester Medical Center, Rochester, NY USA; 4 Department of Medicine, Division of Allergy, Immunology, Rheumatology, University of Rochester Medical Center, Rochester, NY USA

**Keywords:** Inflammation, Arthritis, Lymphatics, Vasculature, Smooth Muscle Cells, Fibroblasts, Monocytes, Macrophages, Single-cell RNA-sequencing, Transcriptomics

## Abstract

**Background:**

Lymphatic dysfunction exists in tumor necrosis factor transgenic (TNF-Tg) mice and rheumatoid arthritis (RA) patients. While joint-draining TNF-Tg popliteal lymphatic vessels (PLVs) have deficits in contractility during end-stage arthritis, the nature of lymphatic muscle cells (LMCs) and their TNF-altered transcriptome remain unknown. Thus, we performed single-cell RNA-sequencing (scRNAseq) on TNF-Tg LMCs in PLVs efferent to inflamed joints versus wild-type (WT) controls.

**Methods:**

Single-cell suspensions of PLVs were sorted for smooth muscle cells (SMCs), which was validated by *Cspg4*-Cre;tdTomato reporter gene expression. Single-cell RNA-seq was performed on a 10x Genomics platform and analyzed using the Seurat R package. Uniform Manifold Approximation and Projections (UMAPs) and Ingenuity Pathway Analysis software were used to assess cell clusters and functional genomics in WT vs. TNF-Tg populations.

**Results:**

Fluorescent imaging of *Cspg4*-Cre;tdTomato vessels demonstrated dim PLVs and strong reporter gene expression in the adjacent superficial saphenous vein, which was corroborated by flow cytometry of LMCs and vascular smooth muscle cells (VSMCs) from these vessels. Due to their unique morphology, these populations could also be readily detected by scatter analysis of cells from non-fluorescent mice. Bioinformatics analysis of flow sorted WT and TNF-Tg cells identified 20 unique cell clusters that together were 22.4% LMCs, 15.0% VSMCs, and 62.6% non-muscle cells of 8879 total cells. LMCs and M2-macrophages were decreased, while inflammatory monocytes were increased in TNF-Tg lower limb vasculature. SMC populations were defined by *Cald1*, *Tpm1*, and *Pdgfrb* expression and were enriched in myofibroblast-like gene expression. TNF-Tg LMCs exhibited enhanced functional genomics associated with cell death, phagocyte recruitment, and joint inflammation. Among the most prominent TNF-induced genes in SMCs were *Mmp3*, *Cxcl12*, and *Ccl19*, and the most downregulated genes were *Zbtb16*, *Galnt15*, and *Apod*.

**Conclusions:**

Single-cell RNA-seq can be used to investigate functional genomics of lower limb vasculature in mice. Our findings confirm the inflammatory transcriptome of TNF-Tg vessels and altered gene expression in SMC populations. This study further supports a potential role of mesenchymal stromal cells in inflammatory-erosive arthritis pathogenesis, and warrants future studies to define the effects of this TNF-altered transcriptome on PLV function and joint homeostasis.

**Supplementary Information:**

The online version contains supplementary material available at 10.1186/s13075-022-02730-z.

## Background

While the mechanisms of adaptive immunity in rheumatoid arthritis (RA) have been extensively studied, stromal cells have recently been recognized as an integral component of inflammatory-erosive arthritis pathogenesis [1, 2]. Specifically, synovial fibroblasts have been demonstrated to mediate a direct role both in the inflammatory (THY1^+^) and erosive (THY1^-^) processes within the joint [3]. Transcriptomic approaches also identified additional subsets of these synovial fibroblasts, and in particular a peri-vascular fibroblast subset that was selectively increased in RA synovium compared to osteoarthritis [4]. In addition, mechanisms have been proposed relating the recently discovered preinflammatory mesenchymal (PRIME) cells shown to be elevated in peripheral blood preceding an arthritic flare, but then decrease dramatically upon flare onset as the cells presumably migrate into the synovium to differentiate into the pathologic fibroblast subsets [5].

Along with these recent advances implicating a direct relationship between mesenchymal cells in inflammatory arthritis, our previous studies have focused on associated mechanisms related to the pathogenesis of synovial and joint-draining lymphatics in RA [6]. In mice, the synovial lymphatics that drain the ankle joint eventually culminate in collecting popliteal lymphatic vessels (PLVs), two of which traverse on either side of the superficial saphenous vein (SSV) and drain directly into the efferent popliteal lymph node (PLN). The collecting PLVs are largely composed of two cell types, lymphatic endothelial cells (LECs) and lymphatic muscle cells (LMCs). The LMCs exhibit the capacity to both provide vessel tone and pump lymph through coordinated contractions [7]. Previous studies in the tumor necrosis factor transgenic (TNF-Tg) mouse model of RA demonstrated that these PLVs are able to initially drain the inflamed joint effectively leading to dramatic PLN expansion with limited joint disease [8, 9]. However, after a prolonged period, the expanded PLN suddenly collapses with loss of fluid flow, which is concomitant with reduced afferent lymph drainage and PLV contractions correlated with the onset of severe synovitis and bone erosions [10–12]. Ex vivo assessment of cannulated wild-type (WT) vs. TNF-Tg PLV function revealed intrinsic defects in contractility isolated from the afferent joint and efferent PLN regardless of PLN expansion or collapse, suggesting that defects in PLV contractility are the inciting event in this process of lymphatic failure and arthritic progression [13]. Importantly, anti-TNF therapy is able to restore these PLV contractions associated with amelioration of arthritis [11]. We have also translated these findings to human RA, where subjects with active arthritis in their hands demonstrated significantly reduced lymphatic clearance from the web spaces adjacent to the metacarpophalangeal joints by near-infrared imaging of indocyanine green [14].

In our recent work, we have thoroughly evaluated the role of nitric oxide (NO) signaling in mediating the defects in lymphatic contractility noted during the progression of inflammatory arthritis. NO formation through inducible NO synthase (iNOS) produced by peri-lymphatic CD11b^+^ myeloid cells [15] and the LECs [16] themselves has been directly implicated to reduce lymphatic contractility with inflammation. In TNF-Tg mice with collapsed PLNs, CD11b^+^ and iNOS producing myeloid cells were found to be stagnant within PLVs by intravital microscopy [17] and progressively adhere to the LECs [11], likely involved in the contractile dysfunction. Importantly, treatment with a selective iNOS inhibitor in vivo was sufficient to recover PLV contraction frequency in TNF-Tg mice [16], and TNF-Tg x iNOS^−/−^ mice exhibited reduced joint disease at early time points in females with accelerated arthritic progression [18, 19]. Detailed assessment of PLV contractility ex vivo revealed that NOS inhibition similarly improved contraction frequency, but was unable to fully recover the reduced contractile amplitude [13]. Together, these studies demonstrated that contractility defects in TNF-Tg PLVs are only partially related to NO and suggested that the LMCs may be directly damaged and/or transcriptionally modified to explain the persistent PLV dysfunction that remained despite NOS inhibition. Thus, we completed the first single-cell RNA-sequencing (scRNAseq) analysis of PLV-LMCs and the vascular smooth muscle cells (VSMCs) of the adjacent SSV from mice, to define the transcriptome of these cell populations based on functional genomics. We also compared the transcriptomes of the identified LMC and VSMC clusters from WT and TNF-Tg mice to gain insights on the prototypic smooth muscle cell (SMC) genes expressed by these cells, and alterations in this homeostatic transcriptome in the setting of chronic TNF-induced inflammatory-erosive arthritis.

## Methods

### Mouse models and treatments

Animal experiments were approved by the University Committee for Animal Resources at the University of Rochester. All mice used for this study were maintained on a C57BL/6J genetic background. For the initial experiments to enrich for PLV-LMCs and SSV-VSMCs by fluorescence activated cell sorting (FACS) of tdTomato (tdT)^+^ cells, male and female *Cspg4*-Cre mice (Jackson Laboratory #029926) [20] were crossed with Ai9-tdTomato reporter animals (Jackson Laboratory #007909) [21], as previously described [22]. *Csgp4*-Cre^+/−^, Ai9-tdTomato^+/−^ offspring > 8 months old were used for the experiments (*n* = 3 mice / experiment, 2 replicates, *n* = 6 total mice), while *Cspg4*-Cre^−/−^, Ai9-tdTomato^+/−^ littermates (*n =* 1 mouse / experiment, *n =* 2 total mice) were used as negative controls to accurately gate for tdT^+^ cells. To evaluate PLV-LMCs and SSV-VSMCs by scRNAseq in inflammatory-erosive arthritis, 8–9-month-old TNF-Tg (3647 line, *n =* 3 mice) and WT mice (*n =* 3 mice) were used. Only male WT and TNF-Tg mice were used for the study given the early mortality of TNF-Tg females [19]. The TNF-Tg mice were originally obtained by Dr. George Kollias [23, 24] and have been maintained at the University of Rochester.

The experiments to validate the scRNAseq findings by whole mount immunofluorescent microscopy were performed in *Pdgfrb*-CreER (Jackson Laboratory #030201) [25], *Acta2*-Cre (Jackson Laboratory #029925) [20], and *Acta2*-CreER (a gift from Dr. Ivo Kaljzic at the University of Connecticut) [26] all crossed into Ai9-tdTomato reporter models. For the CreER experimental mice, tamoxifen (Millipore Sigma Cat# T5648) was dissolved in corn oil (Millipore Sigma Cat# C8267) at 10 mg/mL. In the *Pdgfrb*-CreER mice (2.5 months old), tamoxifen administration was performed by weight at 0.1 mg/g intraperitoneal, while for the adult *Acta2*-CreER (5 months old) a standard volume of 200 μL was injected intraperitoneal for 5 consecutive days. To confirm efficiency of the intraperitoneal tamoxifen administration, a *Pdgfrb*-CreER mouse was also induced via local administration of 10 μL into the footpad to drain directly into the PLVs for 5 consecutive days. Evaluation of tdT^+^ cells after tamoxifen induction was performed at least 7 days after the final injection.

The following primer sequences were used for genotyping of TNF-Tg and Cre lines:

- TNF-Tg Forward: 5′-TAC-CCC-CTC-CTT-CAG-ACA-CC-3′
- TNF-Tg Reverse: 5′-GCC-CTT-CAT-AAT-ATC-CCC-CA-3′
- Cre Forward: 5′-CCT-GGA-AAA-TGC-TTC-TGT-CCG-TTT-GCC-3′
- Cre Reverse: 5′-GAG-TTG-ATA-GCT-GGC-TGG-TGG-CAG-ATG-3′

### Tissue harvest and dissociation

Experimental mice were administered a lethal dose of ketamine / xylazine, and the fur on the hindlimbs was removed with depilatory cream. Evan’s blue dye (2%, Millipore Sigma Cat# E2129) was administered into the hindpaws to be absorbed by the PLVs. Before beginning the dissections, all equipment and gloves were cleaned thoroughly with RNase Zap (Ambion, Cat# AM9780) and 70% EtOH. The hindpaw was then secured to a silicone dish, and an incision was made on the posterolateral surface of the calf. The skin was removed to reveal the two PLVs (blue from the dye) that run parallel to the SSV, as previously described [22]. Forceps were used to grasp all three vessels (two PLVs and the SSV), and the vessels were dissected away from the underlying tissue then stored on ice in 200 μL (0.65 mL Eppendorf tube) of Dulbecco’s modified Eagle’s medium (DMEM) + GlutaMAX (Gibco, Cat# 10566-016) supplemented with 10% fetal bovine serum (FBS; Millipore Sigma Cat# F0926). After the vessels from all the mice were dissected, the vessels were sequentially placed under a microscope in a drop of the supplemented DMEM and the adipose tissue surrounding the vessels was shred by forceps to mechanically release the vessels from the fat, then the tissue was placed back on ice in the 200 μL of media. The bottom and top of a 100 × 15 mm petri dish (Falcon Cat# 351029) were both filled with sterile Dulbecco’s phosphate buffered saline (DPBS; Gibco Cat# 14190-144). The vessels were sequentially washed in the bottom and top of the petri dish, then cut into 4 or more small pieces. The vessels from one mouse (both hindlimbs) were dissociated into single cells together in 1 mL (1.5 mL Eppendorf tube) of Accumax (Innovative Cell Technologies Cat# AM-105) rotating for 1 h at room temperature. A 70 μm MACS SmartStrainer (Miltenyi Biotec Cat# 130-098-462) was placed on a 15-mL tube and wet with 3 mL of 10% FBS diluted in DPBS. The 1 mL of Accumax with suspended cells was then passed through the strainer into the 15-mL tube, and 1 mL of 10% FBS was used to wash the tube used for the enzymatic digestion then similarly passed through the strainer. This process was performed sequentially for all samples for a given experiment, thus pooling all cells from *n =* 3 mice. The cells were then pelleted at 300 g and 4 °C, and the supernatant was carefully removed until approximately 500 μL remained. An additional 500 μL of DPBS was then added and the cells were resuspended. The tube was then lightly vortexed to rinse the sides of the tube, and the 1 mL of total fluid was transferred to a 1.5-mL Eppendorf tube. The cells were then pelleted at 600 g and 4 °C, then resuspended in 500 μL of DPBS. One drop of NucBlue Fixed Cell Stain (DAPI formulation, Thermo Fisher Scientific Cat# R37606) was added to label dead cells. The cells were then transferred to the Flow Cytometry Core (FCC) at the University of Rochester on ice.

### FACS and single-cell library preparation

**Fig. 1:**
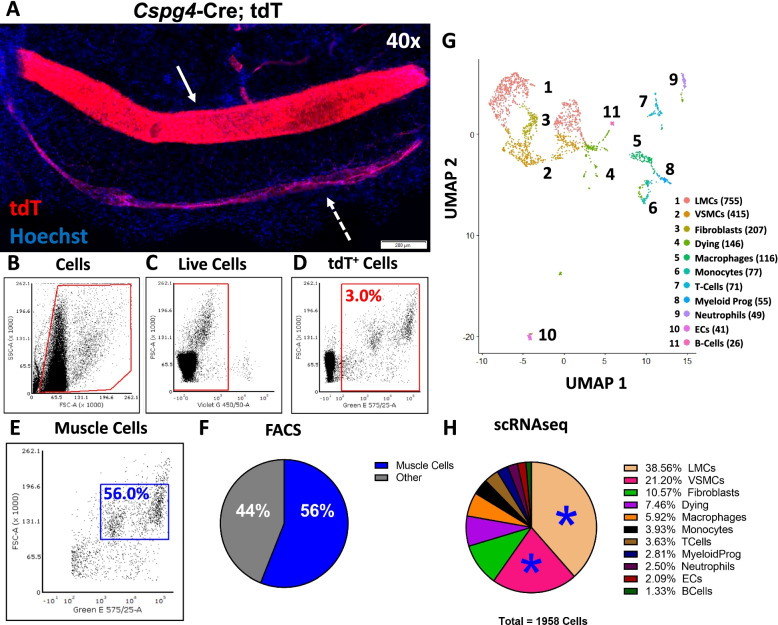
FACS enrichment of tdT^+^ SMCs from lower limb vasculature of Cspg4-Cre;Ai9-tdTomato mice validated by scRNAseq. Popliteal lymphatic vessel **(**PLV) (dashed arrow) and the adjacent superficial saphenous vein (SSV) (solid arrow) from *Cspg4*-Cre;tdTomato (tdT) mice were processed for whole mount fluorescent microscopy [22], and a representative × 40 image is shown (**A**). A single-cell suspension was prepared by pooling the digested SSV and both adjacent PLVs from the hindlegs of *Cspg4*-Cre;tdT mice (*n =* 3), which were sequentially sorted by fluorescence activated cell sorting (FACS) to exclude debris (**B**), and enrich for live (DAPI^−^, **C**) and tdT^+^ cells (**D**). Two replicates (*n =* 3 mice each) were performed with 16,896 (tdT1) and 2771 (tdT2) events sorted for downstream single-cell RNA-sequencing (scRNAseq) (red gates). Within the tdT^+^ events, two predominant cell populations were noted by tdT signal intensity (within blue rectangle in **E**), presumed to be tdT^lo^ lymphatic muscle cells (LMCs) and tdT^hi^ vascular smooth muscle cells (VSMCs) based on the whole mount studies shown in (**A**). These two populations (inside blue rectangle in **E**) account for 56.0% of the sorted cells (9467 events vs 16,896 total tdT^+^ events for tdT1) (**F**). The two tdT replicates were integrated, and UMAP was used to embed the data into 2D space where a minimally supervised shared nearest neighbor (SNN) clustering algorithm resolved 11 clusters. Each cluster was labeled by cell type with number of cells for each cluster in parentheses, and numbers on the UMAP correspond with the cluster order (**G**). Together, the LMC and VSMC populations (blue stars) represent 59.8% of the total sequenced cells, consistent with the FACS analysis shown in (**E** and **F H**)

**Fig. 2:**
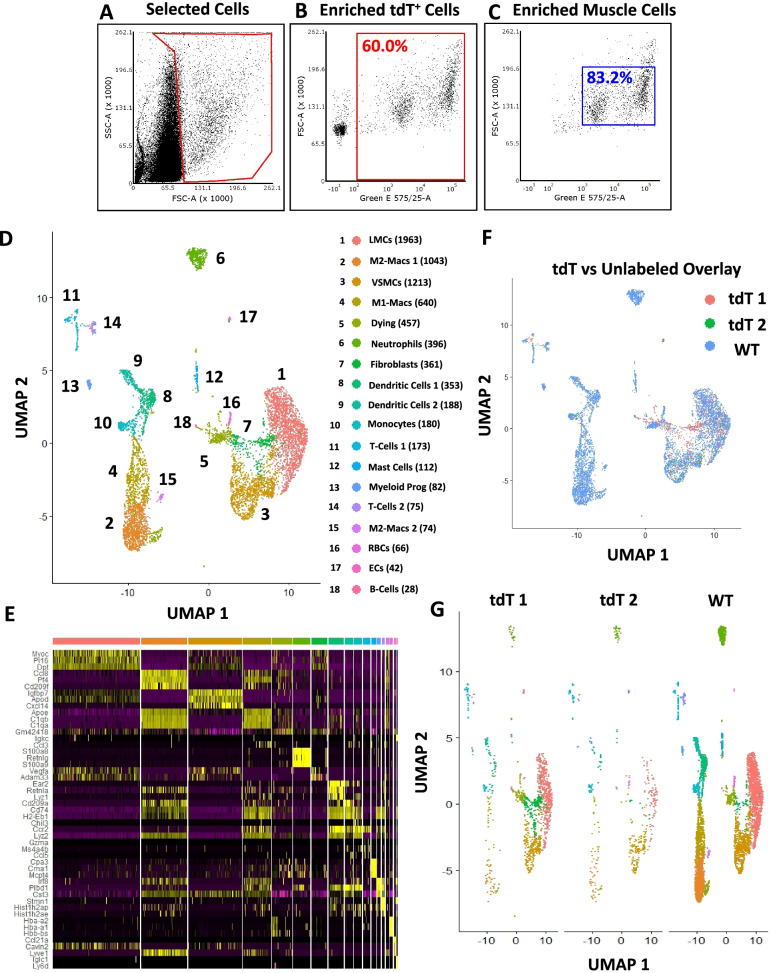
Strategic gating of unlabeled vasculature derived cells from WT mice enriches for SMCs. Further fluorescence activated cell sorting (FACS) analysis of tdTomato (tdT) sorted cells from *Cspg4*-Cre;Ai9-tdT mice described in Fig. 1 revealed that selectively gating for high side and forward scatter events (**A**) enriched for the tdT^+^ smooth muscle cell (SMC) populations, where the percent of tdT^+^ cells relative to total gated live cells increased from 3.0% (gating as in Fig. 1) to 60.0% (**B**). Moreover, 83.2% of these tdT^+^ events represent the SMC populations (relative to 56.0% as in Fig. 1) (**C**). Popliteal lymphatic vessels (PLVs) and superficial saphenous veins (SSVs) from 8–9-month-old wild-type (WT) mice (*n =* 3) were harvested, and the gating strategy represented in **A** was performed and DAPI^−^ live events were selected (13,130 sorted events) for downstream single-cell RNA-sequencing (scRNAseq). The tdT and WT datasets were integrated and a total of 7446 cells (1958 tdT and 5488 WT) were analyzed by minimally supervised shared nearest neighbor (SNN) clustering algorithms in Seurat. Each cluster was labeled by cell type and the total number of cells for each cluster is shown in parentheses (**D**). A heatmap represents the top 3 differentially expressed genes for all 18 cell clusters (duplicate genes omitted) with each cluster color coated and in the same cluster order (left to right) as the UMAP in (**D**) (**E**). A full gene list for each cluster is provided in the Supplementary Materials. Overlay of the two tdT replicates with the WT sample demonstrated remarkable conservation of cell types with limited batch effect (**F**). UMAPs of the tdT replicates and WT sample are shown side-by-side to further depict the conservation of cell types, and proportional changes in cell populations with the two different sorting strategies for tdT and WT samples (**G**)

The day before the experiment, 1.5-mL Eppendorf tubes for cell collection by FACS were filled with 1.5 mL of 100% FBS. Before transfer of the cells to the FCC, the 100% FBS was removed and replaced with 150 μL of 2% FBS diluted in DPBS. Both an 85 μm (tdT1) and 100 μm (tdT2, WT, and TNF-Tg) nozzle was used for the FACS on a BD FACSArea II instrument sorted with a purity mask. The tdT cell samples from the *Cspg4*-Cre;Ai9-tdTomato mice were sorted using the strategy outlined in Fig. 1 for all cells by forward/side scatter, DAPI^−^ live cells, and then tdT^+^ cells as the final gate for collection. The WT and TNF-Tg cell samples were sorted using the gating strategy described in Fig. 2 specific for high forward/side scatter events. These non-fluorescent samples were similarly sorted for DAPI^-^ live cells as the final gate for collection. Importantly, after the first experiment conducted on tdT1, we modified a number of parameters in order to enhance cell viability post-sorting. The following parameters for experiments tdT2, WT, and TNF-Tg were changed: 1) Increased nozzle size from 85μm to 100μm; 2) Reduced the possibility of cell adhesion to the FACS collection tube by filling the collection tube with 100% FBS held at 4°C overnight before replacing with the collection media; and 3) Relied on FACS sorting of DAPI^-^ live cells to quantify viability, and omitted steps to measure viability before sequencing given cell count and time constraints. These parameter modifications may explain the slight difference in events and cell counts among distinct scRNAseq experiments.

The cells were immediately transferred to the Genomics Research Center at the University of Rochester for single-cell library preparation. The sorted cells were counted and then loaded on a Chromium Single-Cell Instrument (10x Genomics, Pleasanton, CA, USA) to generate single-cell Gel Bead-in-Emulsions (GEMs). Library generation for scRNAseq was accomplished using the Chromium Single-Cell 3′ Library & Gel Bead Kit (10x Genomics) based on the manufacturer’s instructions. After reverse transcription of the GEMs, the barcoded cDNA was purified with DynaBeads MyOne Silane Beads (Thermo Fisher Scientific Cat# PN37002D) and amplified by PCR for 3′ cDNA library construction. Illumina’s (San Diego, CA, USA) NextSeq 550 (tdT1 sample) and NovaSeq 6000 (tdT2, WT, and TNF-Tg samples) sequencers were then used to generate the sequenced data. Note, when evaluating the relationship between FACS event and sequenced cell counts, many factors such as adhesion of cells to the collection tube, cell loss when concentrating cells for sequencing, and/or variable single-cell capture efficiency may lead to different total downstream sequenced cell numbers. Nevertheless, our FACS and sequencing strategies provide an optimal condition for enhanced cell viability post-sorting and high single-cell capture efficiency.

### Single-cell RNA-seq data analysis

**Fig. 3:**
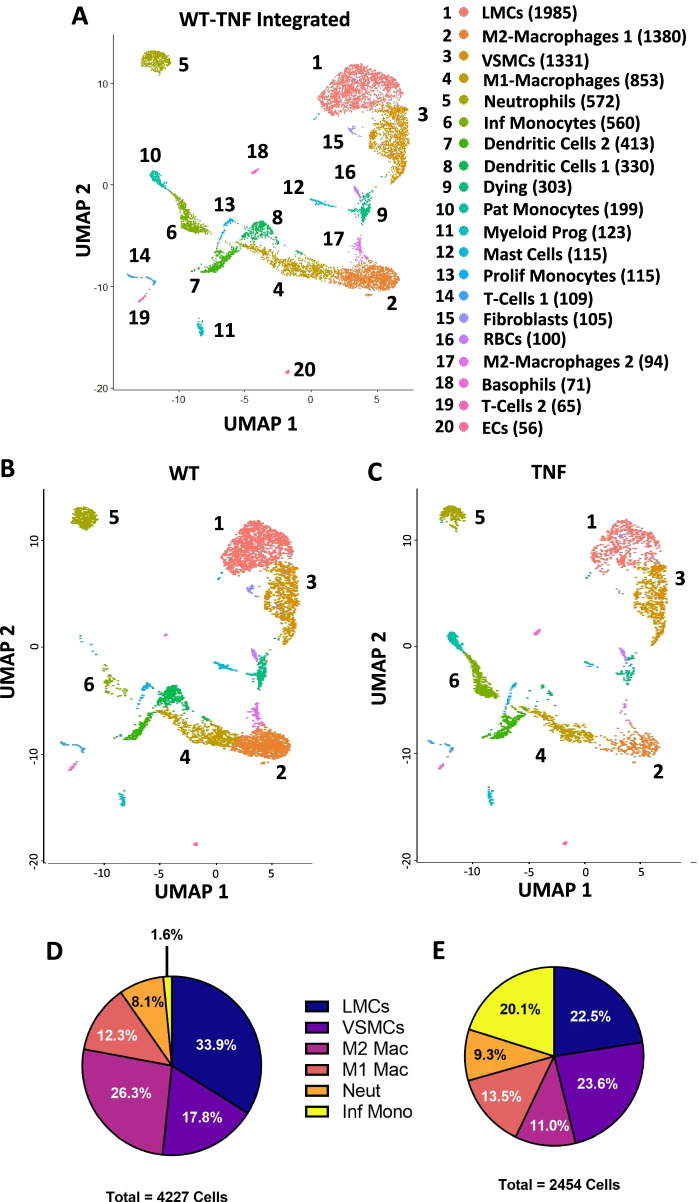
Decreased LMCs and M2-macrophages with increased inflammatory monocytes from peripheral vasculature of TNF-Tg arthritic mice. To compare with the wild-type (WT) dataset described in Fig. 2, popliteal lymphatic vessels (PLVs) and superficial saphenous veins (SSVs) from 8–9-month-old tumor necrosis factor transgenic (TNF-Tg) mice (*n* = 3) with 6963 events were isolated by fluorescence activated cell sorting (FACS) for single-cell RNA-sequencing (scRNAseq). The WT and TNF-Tg datasets were integrated and the unsupervised shared nearest neighbor (SNN) clustering algorithm in Seurat resolved 20 distinct cell clusters from the 8879 total cells (5488 WT and 3391 TNF) analyzed. Each cluster was labeled by cell type with total number of cells for each cluster in parentheses and each cluster numbered on the UMAP (**A**). Sub-analysis of the top 6 most abundant cell populations in the WT (**B**) and TNF-Tg (**C**) samples was performed to assess changes in cell proportions between the conditions. In the 4227 subclustered cells from the WT dataset, a small proportion of the cells represented inflammatory monocytes (1.6%), while lymphatic muscle cells (LMCs) (33.9%) and M2 macrophages (26.3%) were the predominant cell populations (**D**). In the 2454 subclustered cells from TNF-Tg mice, the inflammatory monocyte population expanded dramatically to 20.1%, while both LMCs and M2 macrophages decreased to 22.5% and 11.0%, respectively (**E**). The cell counts and percentages for each cluster between WT and TNF-Tg vasculature are provided in Table 1. Supplementary Figure 4 describes the markers used to define the specific monocyte and macrophage populations, while Fig. 4 and Supplementary Figures 5, 6, and 7 together describe the identification of LMCs

The filtered feature matrices were imported and analyzed using the Seurat packages (Seurat v4.0.3) in RStudio (v1.2.1335; R v4.1.1) [27–30]. A shared nearest neighbor (SNN) clustering algorithm was used to embed the data into 2D space using a Uniform Manifold Approximation and Projection (UMAP) [31]. The data was processed according to the instructions of the Seurat R package and the cell clusters were then defined using resolution = 0.5. For Fig. 3, the clusters were generated in an unsupervised manner, while generation of cell clusters for Figs. 1 and 2 and Supplementary Figure 7 was minimally supervised for comparable clustering of the SMC populations as in Fig. 3. For example, prior to user supervision for Fig. 1, the VSMC population was split into 2 clusters that were then merged together as determined by unsupervised clustering when more cell types were sequenced in Fig. 3. Multiple scRNAseq datasets were integrated using commonly expressed gene anchors, while the identities of the original datasets could be extracted for comparative analysis. The “FindAllMarkers” command was used to identify the differentially expressed genes among each cluster, while “FindMarkers” with identities specified (i.e., WT or TNF-Tg, LMC or VSMC) was used to determine changes in gene expression between groups. These commands calculate the log_2_ fold-change (log2FC) and false discovery rate (FDR) based on statistical analysis using the non-parametric Wilcoxon rank sum test between the specified groups. The R package ggplot2 (v3.3.5) [32] was used to create the violin plots for comparing gene expression.

### Pathway analysis

Differentially expressed genes (DEGs) between TNF-Tg and WT LMCs (Supplementary Materials) were uploaded into the Ingenuity Pathway Analysis (IPA; July 2021 release, Qiagen, Hilden, Germany) software. The “Expression” core analysis was performed based on expression log ratio. Within the core analysis, the graphical summary, canonical pathways, and diseases and functions (inflammatory response) panels were directly evaluated. The graphical summary was provided directly from IPA, while the additional gene expression and pathway graphs were generated in GraphPad Prism (v9.1.0, San Diego, CA, USA) based on exported Excel sheets.

### Immunostaining and fluorescent microscopy

PLVs with or without the adjacent SSV were processed for whole mount immunofluorescent microscopy, as previously described [22]. Briefly, after dissection the vessels were fixed in 10% normalized buffered formalin (NBF) for 30 min, and then washed in 0.1% Triton X-100 (Millipore Sigma Cat# X100) / 1× TBS (Bio-Rad Cat# 1706435) solution (3×, 10min) rocking at room temperature. The vessels were then permeabilized overnight in 0.3% Triton X-100 / 1× TBS solution rocking at 4 °C. The vessels were blocked in 5% normal goat serum (Thermo Fisher Scientific Cat#50062Z) / 0.3% TritonX-100 / 1× TBS solution rocking at room temperature for 1 h. The vessels were incubated overnight rocking at 4 °C in primary antibody solution diluted in 5% normal goat serum / 0.3% TritonX-100 / 1× TBS; anti-alpha smooth muscle actin (αSMA) Alexa Fluor 488 conjugated antibody (Thermo Fisher Scientific Cat# 53-9760-82) was used at 1:100 dilution. To wash the primary antibody, the vessels were incubated with 0.1% TritonX-100 / 1× TBS (3×, 10 min) then mounted on a microscope slide with 1 drop of ProLong Gold Antifade Mountant (Thermo Fisher Scientific Cat# P36930) and NucBlue Live ReadyProbes (Hoechst 33342 formulation; Thermo Fisher Scientific Cat# R37605). The slides were then imaged by an Olympus VS120 slide scanner or Nikon A1R HD confocal microscope.

### Micro-computed tomography (μCT)

After vessel dissection, the ankles were harvested and submitted for μCT at the Biomechanics and Multimodal Tissue Imaging Core in the Center for Musculoskeletal Research at the University of Rochester. The ankle joints were imaged using the Scanco Medical VivaCT 40 with the following imaging parameters: 55 kV, 145 μA, 300 ms integration time, 2048 × 2048 pixels, 1000 projections over 180°, and resolution of 17.5 μm isotropic voxels. The DICOM files were then imported into Amira software (v2020.1, Thermo Fisher Scientific, Hillsboro, OR, USA) and the talus was segmented for bone volume measurements, as previously described [33]. Briefly, a set threshold of 2500 Hounsfield units (HU) was used to define bone, and the talus was segmented in the Segmentation Editor. A “Volume Rendering” module with lookup table set to 2500–4500 HU was used for visualization, while “Generate Surface” and “Surface View” modules were used to overlay the talus segmentation in the image. The “Material Statistics” module on the label field defining the talus was used to extract the volume measurements. The volume measurements were exported to GraphPad Prism for statistical analysis using an unpaired *t*-test to compare WT and TNF-Tg talus bone volumes.

### Statistical analysis

To statistically evaluate changes in gene expression across the scRNAseq experiments, the non-parametric Wilcoxon rank sum test was performed in R between groups with FDR (adjusted *p*-value) of < 0.01 considered significant. All other statistical analysis was performed in GraphPad Prism.

### Data availability

The scRNAseq datasets generated in the current study are deposited on NIH Gene Expression Omnibus (GEO; accession number GSE190999), and source code will be provided upon reasonable request. Excel sheets describing the differentially expressed genes for (1) tdT-WT integrated clusters (Fig. 2), (2) WT-TNF integrated clusters (Fig. 3 and Supplementary Figure 2), (3) LMC-VSMC cluster subsets from WT-TNF integration (Supplementary Figure 7), (4) LMC cluster in the WT vs TNF-Tg condition (Fig. 5, Table 2, and Table 3), and (5) VSMC cluster in the WT vs TNF-Tg condition (Table 2 and Table 3) are provided in the Supplementary Materials. All other datasets generated or analyzed during this study are either included in this published article (and its supplementary information files) or will be made available upon reasonable request.

## Results

### FACS enrichment of tdT^+^ SMCs from lower limb vasculature of Cspg4-Cre;Ai9-tdTomato mice validated by scRNAseq

Based on our recent demonstration of tdT reporter gene expression in LMCs and VSMCs of lower limb vasculature from *Cspg4*-Cre;Ai9-tdT mice [22], we aimed to utilize the reporter gene expression as an approach to isolate primary LMCs and VSMCs for scRNAseq. Fluorescent microscopy of the isolated PLV and adjacent SSV demonstrated that the VSMCs exhibited more intense fluorescence than LMCs (Fig. 1A). Quantification of reporter tdT signal intensity in these vessels confirmed that SSVs were significantly brighter (63.83 ± 5.43 artificial units (AU)) than PLVs (22.28 ± 5.45 AU) (*p* < 0.05 by unpaired *t*-test, *n =* 5 vessels each group). To assess the utility of this fluorescent label for isolation of the SMC populations, we pooled single-cell suspensions from digested PLVs and associated SSVs from *Cspg4*-Cre;Ai9-tdT mice for FACS enrichment of tdT^+^ cells (Fig. 1B–D). Post-sort analysis of the tdT^+^ cells revealed two distinct populations based on tdT expression, which accounted for 56.0% of the sorted cells isolated for scRNAseq (Fig. 1E, F). For validation of internal consistency with this FACS protocol, the tdT sort was performed twice with each replicate referred to as tdT1 and tdT2. The scRNAseq of both tdT replicates was performed separately, and then merged together for analysis on an integrated dataset. After removing low-quality cells, a total of 1958 cells were used for downstream analysis. With a minimally supervised clustering approach (defined in “Methods”), we identified 11 cell populations (Fig. 1G), 3 of which were determined to be stromal cells due to high expression of *Prrx1*; hematopoietic cells were identified by *Ptprc* (CD45) enrichment, while remaining low-quality, dying cells were defined by enhanced mitochondrial gene expression (i.e., *mt-Cytb*) (Supp. Fig. 1). Remarkably, the clusters identified as the SMC populations made up 59.8% of the cells analyzed by scRNAseq (Fig. 1H), similar to the proportion predicted by FACS in Fig. 1F.

### Strategic gating of unlabeled vasculature derived cells from WT mice enriches for SMCs

A serendipitous finding of our initial scRNAseq experiment with *Cspg4*-Cre;Ai9-tdT mice was that the reporter dim (LMC) and bright (VSMC) populations identified by tdT fluorescent signal (Fig. 1D) could also be identified by forward and side scatter signals by flow cytometry. Thus, we performed a post hoc analysis to determine if selecting for high forward and/or high side scatter events outside of the predominant cell conglomerate (Fig. 2A) enriches the tdT^+^ cell population. The results confirmed that the majority (60.0% of total events in red gate) are the tdT^+^ cells we aimed to sort for scRNAseq (Fig. 2B). Furthermore, by selecting for high forward and/or side scatter events, many of the proposed non-SMC events that contaminated the tdT^+^ cells from Fig. 1 were excluded with this approach, increasing the SMC proportion from 56.0% (Fig. 1F) to 83.2% (Fig. 2C) of the tdT^+^ cells sorted in Fig. 1. Based on this analysis, single-cell suspensions from the PLVs and SSVs of 8–9-month-old WT mice (*n =* 3) were sorted by FACS solely based on these high forward and/or side scatter parameters, and scRNAseq was performed on the sorted cells. For scRNAseq analyses, the 7446 total cells from the two tdT replicates and the WT sample were integrated to evaluate conservation of the cell types (Fig. 2D), which identified 18 unique cell clusters defined by their differential gene expression (Fig. 2E). When the datasets were overlayed (Fig. 2F) and shown side-by-side (Fig. 2G), there was remarkable consistency of the cell types between the samples. As further demonstration of the selectivity for tdT expression in the stromal cells, there was a dramatic increase in hematopoietic cell populations (predominately monocytes, macrophages, and neutrophils) for the WT sample, which were relatively sparse in the tdT replicates. However, with the gating strategy shown in Fig. 2A, the SMCs remained a predominant cell population. In fact, the selectivity of SMCs for high forward (large) and/or high side (irregular shape) scatter events is intuitive based on the known large and elongated cell shape of SMCs.

### Decreased LMCs and M2-macrophages with increased inflammatory monocytes from joint-draining vasculature of TNF-Tg arthritic mice

Based on the success of the sorting strategy introduced in Fig. 2, we repeated this process for 8–9-month-old TNF-Tg mice (*n =* 3). Integration of PLVs and SSVs from WT and TNF-Tg mice identified 20 unique cell clusters from a total of 8879 cells (5488 WT and 3391 TNF-Tg) analyzed (Fig. 3A) with differentially expressed genes for each cluster depicted as a heatmap (Supp. Fig. 2). The 8–9-month-old TNF-Tg mice used in this study were confirmed to have severe arthritis in their ankles afferent to the PLVs by μCT with significantly reduced talus bone volumes (WT 1.39 ± 0.07 vs TNF-Tg 0.49 ± 0.12 mm^3^, *p* < 0.0001; Supp. Fig. 3). The various monocyte and macrophage populations were identified as a group based on *Itgam* (CD11b) expression, and subdivided based on gene enrichment, where patrolling monocytes (Pat Monocytes, cluster 10) express *Cx3cr1*, inflammatory monocytes (Inf Monocytes, cluster 6) express *Ccr2* and *Ly6c2*, and the primary M2-macrophage population (M2-Macrophages 1, cluster 2) was defined as *Cd163*^Hi^/*Mrc1*^Hi^/*Lyve1*^Hi^ relative to the *Ccr2*^+^/*Ccl2*^Hi^ M1-macrophage cluster (cluster 4) [34–37]. Of note was the enrichment of M2-macrophage populations for *Lyve1*, previously identified as an essential factor in mediating collagen content in VSMCs [38] (Supp. Fig. 4). The UMAPs of the WT (Fig. 3B) and TNF-Tg (Fig. 3C) cells analyzed by scRNAseq are shown with the top 6 clusters by number of cells identified to evaluate changes in cell proportions between WT and TNF-Tg mice (Fig. 3D, E). Notably, inflammatory monocytes (cluster 6) expanded dramatically from 1.6% in WT mice to 20.1% in TNF-Tg mice. In addition, the LMC population decreased in TNF-Tg mice from 33.9% in WT to 22.5% in TNF-Tg, similar to recent reports of a progressive reduction of LMC coverage surrounding synovial lymphatic vessels of TNF-Tg animals [39]. Associated with these changes was a considerable decrease in M2-macrophages from 26.3% in WT to 11.0% in TNF-Tg mice, which may negatively impact the homeostasis of LMCs. Changes in all 20 identified cell clusters between WT and TNF-Tg mice are provided in Table 1.

**Table 1:** Changes in cell clusters from the peripheral vasculature between WT and TNF-Tg mice by scRNAseq. The cluster numbers and cell types are aligned with those defined in Fig. 3. The cell numbers associated with each cluster are provided for both wild-type (WT) and tumor necrosis factor transgenic (TNF-Tg) groups along with the total cell counts for each cluster when these datasets are integrated together. To examine the changes in cell populations between the WT and TNF-Tg conditions, a percentage for each cell type was calculated by the number of cells in an individual cluster divided by the total cells within a single group (WT or TNF-Tg)

Cluster	Cell Type	WT #	WT %	TNF #	TNF %	Total #
1	LMCs	1433	26.11	552	16.28	1985
2	M2-Macrophages 1	1111	20.24	269	7.93	1380
3	VSMCs	751	13.68	580	17.10	1331
4	M1-Macrophages	522	9.51	331	9.76	853
5	Neutrophils	344	6.27	228	6.72	572
6	Inf Monocytes	66	1.20	494	14.57	560
7	Dendritic Cells 1	152	2.77	261	7.70	413
8	Dendritic Cells 2	306	5.58	24	0.71	330
9	Dying Cells	236	4.30	67	1.98	303
10	Pat Monocytes	7	0.13	192	5.66	199
11	Myeloid Progenitors	74	1.35	49	1.45	123
12	Mast Cells	98	1.79	17	0.50	115
13	Prolif Monocytes	70	1.28	45	1.33	115
14	T-Cells 1	45	0.82	64	1.89	109
15	Fibroblasts	75	1.37	30	0.88	105
16	RBCs	54	0.98	46	1.36	100
17	M2-Macrophages 2	80	1.46	14	0.41	94
18	Basophils	7	0.13	64	1.89	71
19	T-Cells 2	31	0.56	34	1.00	65
20	Endothelial Cells	26	0.47	30	0.88	56
	Total	5488	100	3391	100	8879

### Prototypic SMC markers *Cald1*, *Tpm1*, and *Pdgfrb* detected in LMCs and VSMCs by scRNAseq

**Fig. 4:**
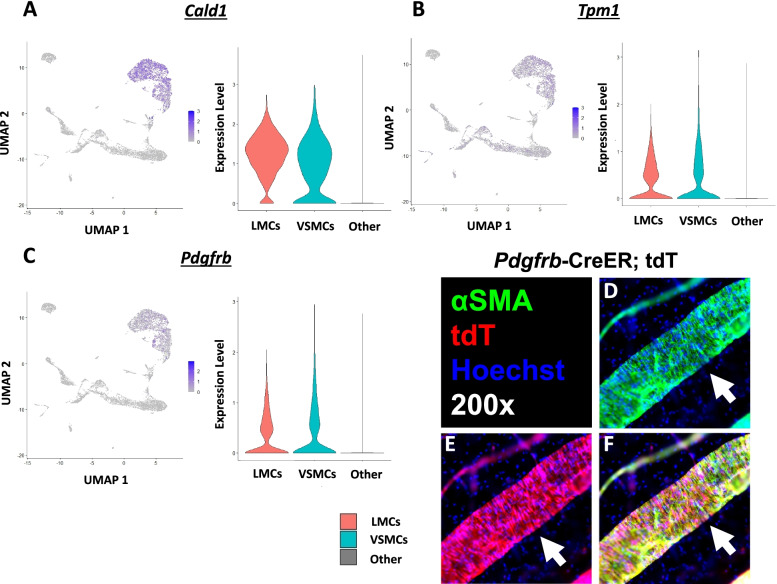
Prototypic SMC markers *Cald1*, *Tpm1*, and *Pdgfrb* detected in LMCs and VSMCs by scRNAseq. To validate the single-cell RNA-sequencing (scRNAseq) clusters predicted to be lymphatic muscle cells (LMCs) and vascular smooth muscle cells (VSMCs) via UMAP analysis, we mined the transcriptomic data for prototypic markers of smooth muscle cells (SMCs). The results showed that *Cald1* (Caldesmon; LMCs 1.77 log_2_ fold-change (log2FC) and VSMCs 1.48 log2FC) (**A**), *Tpm1* (Tropomyosin alpha-1; LMC 0.33 log2FC and VSMC 0.42 log2FC) (**B**), and *Pdgfrb* (platelet-derived growth factor receptor beta; LMCs 0.57 log2FC and VSMCs 0.74 log2FC) (**C**), were significantly enriched relative to all the other clusters. Feature plots overlaying the UMAP are provided to visualize the change in expression (grey = low, blue = high gene expression), while violin plots are shown to demonstrate the effect size between muscle cell populations compared to all other cell clusters. Wilcoxon rank sum test was performed between the different cell types for statistical analysis with false discovery rate (FDR) < 1.01E**−**66 for all comparisons. Sample sizes: LMCs = 1985 cells, VSMCs = 1331 cells, Other = 5563 cells. To validate the expression of *Pdgfrb* in LMCs in vivo, *Pdgfrb*-CreER;tdT mice were generated and induced with tamoxifen. The popliteal lymphatic vessels (PLVs) (solid arrow) were harvested from these animals and immunostained for alpha smooth muscle actin (αSMA) (green) to label LMCs (**D**) and robust tdTomato (tdT) expression (red) was noted in PLVs representing *Pdgfrb* expression (**E**), which colocalized (yellow) with the αSMA^+^ LMCs (**F**)

To validate the identification of the SMC clusters, various evaluations of gene enrichment were performed. As expected, the SMC clusters were significantly enriched for *Cald1* (Caldesmon; Fig. 4A) [40] and *Tpm1* (Tropomyosin alpha-1; Fig. 4B) [41] as typical markers for SMCs [42]. In addition, the SMC and pericyte marker *Pdgfrb* (Platelet-derived growth factor receptor beta) [43, 44] was significantly expressed in the SMC populations (Fig. 4C). To validate enrichment of *Pdgfrb* expression in the SMCs of these vessels, we generated *Pdgfrb*-CreER;Ai9-tdT lineage tracing models. After induction with tamoxifen, the SMCs of harvested PLVs and SSVs demonstrated robust tdT expression that colocalized with αSMA (Fig. 4D–F), as expected from the gene enrichment by scRNAseq. Furthermore, the SMC populations were assessed for lymphangiogenic and angiogenic factors, such as *Vegfa*, *Vegfd*, and *Angpt1* [45, 46], which were all significantly increased in the SMC populations. A combination of muscle-related transcription factors (*Prrx1*, *Twist1*, *Tead1*, *Meox2*, *Id3*, and *Csrp2*), with a role in regulating expression of smooth muscle cell proteins involved in structure and contractility, were also significantly expressed in the SMC clusters. Although none of these transcription factors are specific to SMCs, their combinatorial expression suggests SMC identity (Supp. Fig. 5) [47]. The SMC populations were also directly assessed for common pericyte markers *Cspg4*, *Mcam*, and *Ngfr* [48] all of which were nearly undetectable throughout the dataset. Note that the *Cspg4*-Cre;tdT model used to initially sort SMCs in Figs. 1 and 2 does not presume active expression of *Cspg4* in these cells. Indeed, a previous study reported that after early development, limited or absent *Cspg4* expression was observed in VSMCs and LMCs using inducible *Cspg4*-CreER;tdT models [22]. However, non-specific fibroblast genes *Dcn*, *Fbln2*, and *Pdgfra* [49] were highly expressed in the SMC clusters, which suggests that the PLV-LMCs and SSV-VSMCs are myofibroblast-like (Supp. Fig. 6). To differentiate LMCs from VSMCs, known factors produced by VSMCs such as *Cst3* (Cystatin C/3; 2.60 log_2_ fold-change (log2FC)) [50], *Mgp* (Matrix Gla protein; 2.75 log_2_FC) [51, 52], and *Bgn* (Biglycan; 2.11 log_2_FC) [53, 54] were significantly increased in VSMCs compared to LMCs. Moreover, expression of *Ackr3* (Atypical chemokine receptor 3, formerly CXCR7; 1.39 log_2_FC), known to be selective by *Ackr3*^GFP^ nuclear reporter expression for lymphatic compared to blood vessels in a study specifically investigating the role and noted expression of *Ackr3* in LECs [55], was significantly increased in the LMC cluster (*p* < 2.53E-227 by Wilcoxon rank sum test for all comparisons) (Supp. Fig. 7). Compared to the VSMCs, *Vegfd* was also significantly increased in the LMC cluster and VEGFD is known to interact specifically with VEGFR3 in mice to promote lymphangiogenesis [56, 57]. However, expression of *Vegfc* was nearly undetectable throughout the dataset (Supp. Fig. 5).

However, despite expression of these prototypic markers in the SMC clusters, some conventionally muscle-associated genes were not detected in these SMC populations. For example, canonical markers such as *Acta2* (αSMA), *Myh11* (myosin heavy-chain 11), and *Tagln* (transgelin) [58] showed limited expression throughout the dataset. Given this unexpected result, we generated two different *Acta2* reporter models, *Acta2*-Cre;Ai9-tdT and *Acta2*-CreER;Ai9-tdT mice. Interestingly, tdT expression denoting *Acta2* transcription in vivo was also unexpectedly low in PLV-LMCs and SSV-VSMCs in both of these reporter lines, despite robust protein expression of αSMA (Supp. Fig. 8). In addition, the *Acta2*-CreER;Ai9-tdT mice are reported to exhibit endogenous Cre activation in cells with high αSMA expression, such as the VSMCs of the aorta [26]. However, even after formal induction with intraperitoneal tamoxifen, the tdT expression was limited in the peripheral PLV-LMCs and SSV-VSMCs, suggesting lower *Acta2* expression than VSMCs surrounding large arteries that exhibit endogenous tdT expression even without tamoxifen induction. Taken together, we found the SMC populations to be best identified by expression of various myofibroblast genes, SMC-related transcription factors, and lymphangiogenic or angiogenic factors, rather than prototypic SMC genes.

### TNF-Tg LMCs exhibit gene expression changes associated with known pathways of joint inflammation and arthritis

**Fig. 5:**
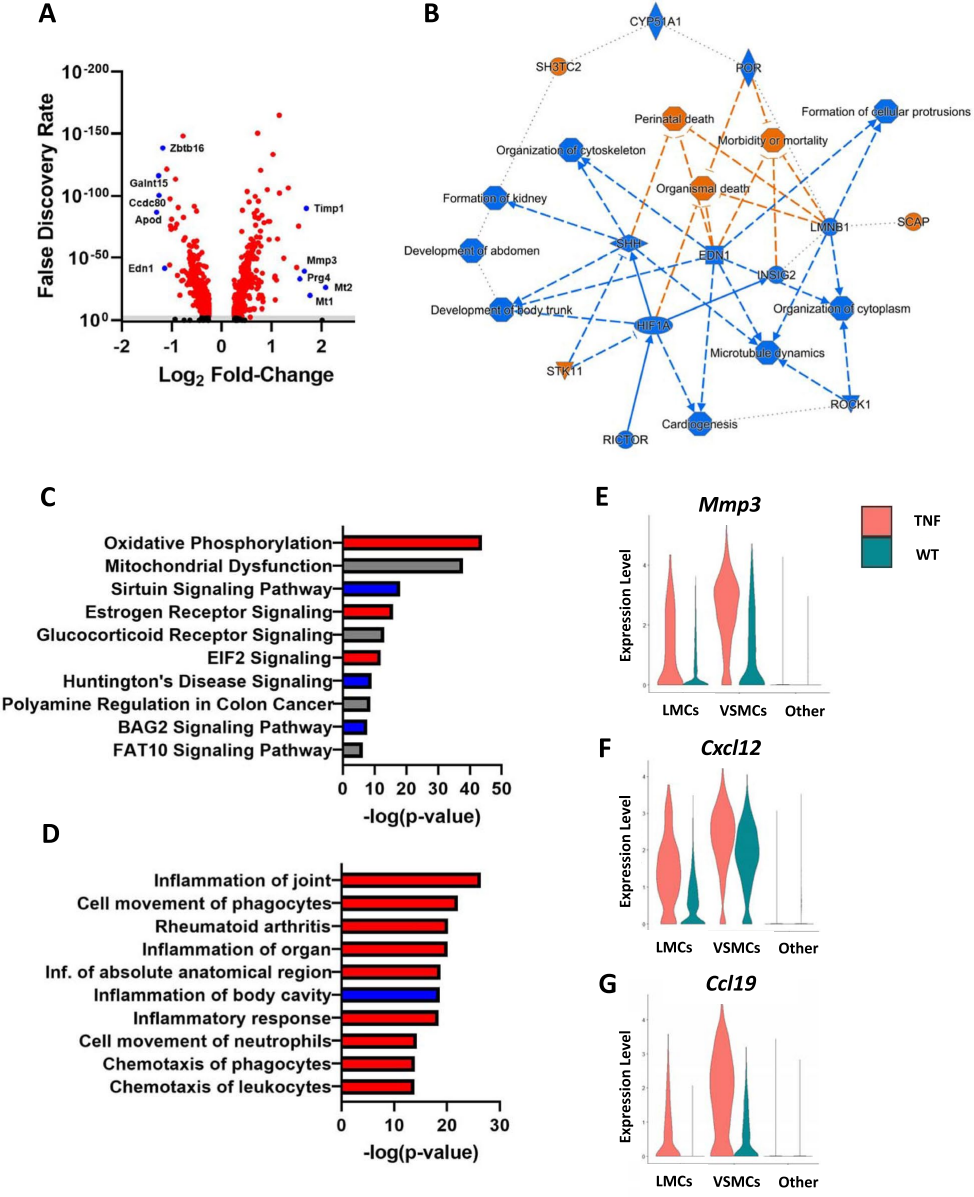
TNF-Tg LMCs exhibit gene expression changes associated with known pathways of joint inflammation and arthritis. Differentially expressed genes of the lymphatic muscle cell (LMC) cluster (cluster 1 in Fig. 3) from wild-type (WT) vs tumor necrosis factor transgenic (TNF-Tg) mice are shown as a volcano plot (**A**). Each dot represents a single gene, and black dots showed no significant change from WT (false discovery rate (FDR) > 0.01, below grey line). The top 5 downregulated (fold-change < 0) and top 5 upregulated (fold-change > 0) in TNF-Tg LMCs are identified by blue dots with gene identifiers. Ingenuity pathway analysis (IPA) was performed on all genes represented in (**A**), and notably the gene pathway modifications (blue = decreased, orange = increased pathway score) ultimately centered on an increase in “Organismal death” (**B**). The top 10 most significantly modified canonical pathways (**C**) and associated inflammatory diseases (**D**) for TNF-Tg vs WT LMCs were identified, where trends of increased (red), decreased (blue), and unchanged (grey) activity patterns are shown. Specific gene expression patterns in TNF-Tg LMCs were identified for their significant involvement in the “Inflammation of joint” pathways, and representative genes most differentially expressed only in the LMC and vascular smooth muscle cell (VSMC) clusters are indicated: *Mmp3* (LMCs 1.65, VSMCs 1.41 log_2_ fold-change (log2FC) in **E**), *Cxcl12* (LMCs 1.54, VSMCs 0.69 log2FC in **F**), and *Ccl19* (LMCs 1.24, VSMCs 2.12 log2FC in **G**). Wilcoxon rank sum test was performed between the same cell types of WT and TNF datasets with an FDR < 4.77E**−**21 for all comparisons. Sample sizes: WT LMCs = 1433 cells, TNF LMCs = 552 cells, WT VSMCs = 751 cells, TNF VSMCs = 580 cells

To further evaluate changes in PLV-LMC gene expression in TNF-Tg compared to WT mice, we further investigated LMCs (cluster 1 in Fig. 3) between the conditions. Log_2_FC was plotted against the FDR to generate a volcano plot showing the most significantly upregulated and downregulated genes where the top 5 genes are annotated for each direction (Fig. 5A). To further highlight the top upregulated and downregulated genes in TNF-Tg SMCs, those genes modified in both LMCs and VSMCs are provided in Table 2, while those specific to LMCs are shown in Table 3. Notably, *Edn1* (Endothelin 1), involved in maintenance of vascular tone and inhibiting SMC apoptosis [59], was dramatically downregulated in TNF-Tg LMCs. *Zbtb16*, a transcription factor previously shown to be downregulated in osteoarthritic cartilage [60], decreased to nearly undetectable levels in TNF-Tg LMCs (WT 85.6% vs TNF-Tg 21.9% of cells expressed the gene with -1.18 log_2_FC). Related to established changes in synovial fibroblast-like populations in inflammatory arthritis, *Thy1* (CD90) demonstrated a significant decrease specifically in TNF-Tg LMCs (Table 3). Interestingly, this finding is consistent with the observation that THY1^−^ fibroblasts are known to be directly involved in bone and cartilage erosions during inflammatory arthritis [3]. Moreover, *Mmp3* has been shown to be significantly increased in TNF-Tg mice [24] and RA patients [61], which serves as important validation of previously established transcriptional changes in inflammatory arthritis. *Prg4* (proteoglycan 4 / lubrican), typically involved in maintaining synovial fluid and tissue homeostasis [62], was also one of the top genes significantly upregulated in TNF-Tg LMCs. While the function of *Prg4* expression in PLV-LMCs is unclear, changes in synovial LMCs may mirror these transcriptional modifications, and ought to be investigated in future studies.

**Table 2:** Differentially regulated genes in TNF-Tg vs WT mice common between LMCs and VSMCs. *Mt2* (and *Mt1*, omitted for redundancy), *Timp1*, *Mmp3*, *Prg4*, and *Cxcl12* are the top 5 upregulated genes in tumor necrosis factor transgenic (TNF-Tg) lymphatic muscle cells (LMCs) that similarly demonstrate increased expression in TNF-Tg vascular smooth muscle cells (VSMCs) (bold). On the other hand, *Apod*, *Galnt15*, *Ccdc80*, *Zbtb16*, and *Klf9* are the top 5 downregulated genes in TNF-Tg LMCs that were also differentially expressed in TNF-Tg VSMCs (not bold). Proteins coded by the gene, log_2_ fold-change (log2FC), and false discovery rates (FDRs) for LMCs and VSMCs are provided. The top 5 genes were ranked based on log2FC effect size with an FDR < 0.01. The genes were selected by evaluating the top up- or downregulated genes for LMCs and listed if the particular genes were also differentially regulated in VSMCs. The LMC and VSMC populations were defined based on the wild-type (WT) and TNF-Tg integrated datasets in Fig. 3. A complete list of differentially expressed genes in TNF-Tg mice for both LMC and VSMC populations is provided in the Supplementary Materials

Gene	Protein	Avg Log_2_FC (LMC & VSMC)	FDR (LMC & VSMC)
***Mt2***	**Metallothionein 2**	**2.08 & 1.19**	**2.26E−20 & 1.04E−13**
***Timp1***	**Timp1**	**1.69 & 1.61**	**7.79E−91 & 1.59E−65**
***Mmp3***	**Matrix Metalloproteinase-3**	**1.65 & 1.41**	**5.58E−40 & 1.27E−61**
***Prg4***	**Proteoglycan 4 / Lubrican**	**1.56 & 1.46**	**7.71E−34 & 1.08E−43**
***Cxcl12***	**C-X-C Motif Chemokine 12**	**1.54 & 0.69**	**2.59E−76 & 4.77E−21**
*Apod*	Apolipoprotein D	**−** 1.30 & **−** 1.61	1.21E**−**87 & 3.99E**−**87
*Galnt15*	Polypeptide N-acetylgalactosaminyl-transferase 15	**−** 1.26 & **−** 1.28	5.5E**−**117 & 4.11E**−**62
*Ccdc80*	Coiled-coil Domain-Containing Protein 80	**−** 1.25 & **−** 1.69	3.8E**−**101 & 1.95E**−**82
*Zbtb16*	Zinc Finger and BTB Domain-Containing Protein 16	**−** 1.17 & **−** 1.07	2.9E**−**139 & 1.84E**−**75
*Klf9*	Krueppel-like Factor 9	**−** 1.10 & **−** 1.08	2.8E**−**122 & 1.62E**−**57

**Table 3:** Differentially regulated genes in TNF-Tg vs WT mice specific to LMCs. *Lbp*, *Mcoln2*, *Slc16a2*, *Adamts1*, and *Calr* are the top 5 upregulated genes that are specific to tumor necrosis factor transgenic (TNF-Tg) lymphatic muscle cells (LMCs) without notable differential gene expression in TNF-Tg vascular smooth muscle cells (VSMCs) (bold). Similarly, *Edn1*, *Thy1*, *Fbn1*, *Cyp2f2*, and *Clic5* are the top 5 genes specifically downregulated in TNF-Tg LMCs (not bold). Proteins coded by the gene, log_2_ fold-change (log2FC), and false discovery rates (FDRs) are provided. The top 5 genes were ranked based on log2FC effect size with an FDR < 0.01. The genes were selected by evaluating the top up- or downregulated genes for LMCs and listed if the particular genes were unique to LMCs and not also differentially regulated in VSMCs. The LMC and VSMC populations were defined based on the wild-type (WT) and TNF-Tg integrated datasets in Fig. 3

Gene	Protein	Avg Log_2_FC	FDR
***Lbp***	**Lipopolysaccharide-Binding Protein**	**0.79**	**3.05E−31**
***Mcoln2***	**Mucolipin-2**	**0.47**	**1.03E−46**
***Slc16a2***	**Monocarboxylate Transporter 8**	**0.46**	**1.14E−30**
***Adamts1***	**A Disintegrin and Metalloproteinase with Thrombospondin Motifs 1**	**0.46**	**3.81E−08**
***Calr***	**Calreticulin**	**0.45**	**6.97E−22**
*Edn1*	Endothelin-1	**−** 1.14	2.45E**−**42
*Thy1*	Thy-1/CD90	**−** 0.69	4.05E**−**29
*Fbn1*	Fibrillin-1	**−** 0.64	2.14E**−**33
*Cyp2f2*	Cytochrome P450/Family 2/Subfamily F/Polypeptide 2	**−** 0.57	1.48E**−**12
*Clic5*	Chloride Intracellular Channel Protein 5	**−** 0.54	4.16E**−**38

To perform a functional genomic analysis on the scRNAseq data, the genes with differential gene expression in TNF-Tg LMCs from Fig. 5A were imported into IPA software, and pathway analysis was performed. A graphical summary of the most significantly modified pathways demonstrated downregulation of the pathways that typically inhibit “Organismal death,” “Morbidity or mortality,” and “Perinatal death,” likely leading to the decreased number of TNF-Tg LMCs noted in Fig. 3 (Fig. 5B). Changes in canonical pathways were evaluated where “Oxidative phosphorylation” and “Mitochondrial dysfunction” showed the most significant overlap with differentially expressed genes in TNF-Tg mice, which may exemplify a compensatory response to the ongoing cellular damage (Fig. 5C). The inflammatory diseases associated with the gene expression changes were also identified, and the most significantly related conditions were “Inflammation of joint,” “Cell movement of phagocytes,” and “Rheumatoid arthritis.” This pathway overlap supports the expected inflammatory arthritic phenotype in TNF-Tg mice, and also the increase of inflammatory monocytes determined in Fig. 3 (Fig. 5D). Representative genes *Mmp3* (Fig. 5E), *Cxcl12* (Fig. 5F), and *Ccl19* (Fig. 5G) were significantly increased in TNF-Tg mice, and associated with the pathway modifications noted in TNF-Tg LMCs, all with known involvement in inflammatory arthritis and/or phagocyte chemotaxis [61, 63, 64]. Altogether, these findings both validate and identify the transcriptional changes in TNF-Tg LMCs, which may be related to the progression of arthritis via various mechanisms.

## Discussion

Although lymphatic dysfunction during arthritic progression is well-recognized, the nature of LMCs that are responsible for LV contractions, and how LMCs change during chronic inflammatory arthritis, particularly at the single-cell level, is largely unknown. As new technologies in scRNAseq have enabled many new insights in the arthritis field [65–70], we aimed to use this platform to elucidate the LMC transcriptome and functional genomics involved in joint homeostasis and advanced disease. While the methods for performing scRNAseq at the commencement of this research existed, a major limitation was the means to isolate viable SMCs from PLV-LMCs and SSV-VSMCs. Indeed, this proved to be the most challenging part of our work, as our initial approach focused on recovering large numbers of LMCs following FACS by pooling PLV-LMCs and SSV-VSMCs from several animals (*n* > 6 mice) proved to be ineffective due to the loss of viability of the sorted cells. In the end, we succeeded by harvesting PLV-LMCs and SSV-VSMCs from only three mice at a time, which reduced the ex vivo manipulation time to < 4 h. While this limits the statistical power of individual experiments that can only study approximately 1000–3000 SMCs per scRNAseq, we found replicate scRNAseq experiments to be highly reproducible such that data could be combined in silico to achieve the desired statistical power.

We were also able to utilize fluorescent reporter models of the peripheral SMCs, and through post hoc analysis of the FACS, generate a gating strategy to enrich for SMCs based on the scatter profile of these cells. This approach demonstrated remarkable conservation of the cell types sequenced after fluorescent enrichment for SMCs, and in turn also provided the opportunity for quantitative analysis of crucial peri-vascular immune cell populations that are integral to vascular function between WT and TNF-Tg conditions. However, one of the primary limitations of sorting based solely on scatter is the possibility for batch effect between the experiments as fluorescent markers were not used for specific cell isolation. Given the time constraints previously noted, each scRNAseq experiment was performed on separate days. To reduce the batch effect, especially between the WT and TNF-Tg groups, the exact same sort conditions and gating were performed. The side-by-side comparisons and conservation of cell types when the scRNAseq datasets were integrated in Figs. 2 and 3 suggest limited batch effects in the sequencing process itself.

Here we demonstrated an increase in inflammatory monocytes along with a reduction in LMC and M2-macrophage populations in joint-draining vascular tissues from mice with advanced inflammatory-erosive arthritis. Moreover, TNF-Tg LMCs exhibited significant transcriptomic changes from WT, which were associated with cell death, phagocyte recruitment, joint inflammation, and RA pathogenesis. Together, this study establishes our first understanding of the transcriptional modifications in TNF-Tg LMCs to guide further research on the role of lymphatic contractility in chronic inflammatory arthritis.

The discovery of changes in peri-vascular immune cell populations and reduced LMCs by scRNAseq both corroborates our past work and provides novel hypotheses to further understand the lymphatic pathology in TNF-Tg mice. In fact, recent studies have implicated a direct role for TNF in the apoptosis of LMCs and reduced contractile gene expression, such as *Acta2*, in synovial lymphatics [39]. Moreover, inflammatory monocytes may be directly recruited to the outside of the vessel by inflamed PLV-LMCs and SSV-VSMCs through increased expression of chemokines, such as *Cxcl12*, and the accumulation of inflammatory monocytes in the surrounding tissue could reduce lymphatic contractility in TNF-Tg mice through iNOS-mediated mechanisms [15]. The accumulation of these peri-vascular inflammatory monocytes may in part explain the effective restoration of lymphatic function with iNOS inhibition in TNF-Tg mice [16]. Along with increased monocyte recruitment, further study into the permeability of inflamed PLVs in TNF-Tg mice is warranted. Collecting lymphatic vessels have been shown to be a point of interaction between permeable lymph contents and surrounding antigen-presenting cells embedded in the adipose tissue that then migrate to draining lymph nodes [71], and may partly explain the dramatic lymph node expansion previously characterized in TNF-Tg mice [9]. The evaluation of peri-lymphatic immune cells involved in negative regulation of lymphatic contractility and LMC homeostasis in TNF-Tg mice, such as inflammatory monocytes/macrophages and mast cells [72–74], is an active area of investigation. The reduction in M2-macrophages with *Lyve1* expression was also previously unappreciated in the TNF-Tg model, and this novel finding is of particular importance given the recently discovered role for peri-vascular *Lyve1* expressing M2-macrophages in regulating homeostatic collagen levels in VSMCs, necessary for proper function [38]. In fact, the decrease in M2-macrophages associated with the well-established reduction in TNF-Tg LMC function may be closely related, and ought to be investigated in future studies. Thus, the scRNAseq both validates and provides further understanding of the pathologic mechanisms associated with lymphatic dysfunction in TNF-Tg mice.

However, despite these key findings in our single-cell analysis of the peripheral vasculature, one of the primary limitations of this study is the reliance on scRNAseq to evaluate quantitative and transcriptomic cellular changes. While we were able to identify distinct changes in cell numbers and transcriptomic modifications in TNF-Tg mice, we were unable to elucidate the spatial relationships, cellular interactions, or functional consequences of these changes solely using scRNAseq. For example, although the reduced LMC population in TNF-Tg mice is similar to findings in synovial lymphatics [39], studies of LMC coverage in the collecting PLVs during inflammatory arthritis are still ongoing. In addition, considerable investigation is necessary to determine whether peri-vascular *Lyve1*^+^ M2-macrophages localize around PLVs and regulate LMC homeostasis similar to VSMCs [38], and whether the reduction of this cell population is associated with lymphatic pathology in TNF-Tg mice. Future studies are needed to comprehensively evaluate the phenotypic switch towards inflammatory monocytes in these peri-vascular regions and the impact of these microenvironmental changes on the function of TNF-Tg PLVs. Thus, the current work has generated valuable hypotheses, but will require further validation and investigation in future studies to elucidate the relationship of our findings to the pathology of inflammatory-erosive arthritis.

An unexpected finding of this study was the limited detection of canonical markers for SMCs in the peripheral vasculature of the lower limb. Since RT-PCR approaches have previously demonstrated that LMCs derived from mesenteric and thoracic lymphatic vessels express *Acta2* (αSMA) and *Myh11* (myosin heavy-chain 11) [75], we expected these markers to be highly enriched in the SMC populations. However, we further validated the low *Acta2* expression by two different Cre models, *Acta2*-Cre [20] and *Acta2*-CreER mice [26], both crossed into the faithful Ai9-tdTomato reporter line [21, 22, 26] with limited tdT expression in PLV-LMCs and SSV-VSMCs.

Moreover, while scRNAseq via droplet-based platforms provides the benefit of high-throughput sequencing, it has been well-established that read depth with this technology is much lower than alternative plate-based approaches, such as Smart-seq2, and thus low frequency transcripts may be dropped in the current study [76]. For example, in a recent study directly comparing 10x and Smart-seq2 expression patterns by Wang et al. [76], cells derived from a liver tumor were processed and 10x concluded that markers of VSMC contraction in the KEGG pathway were undetectable, while Smart-seq2 identified a significant enrichment of this pathway in the same cell types. Similarly, in a recent study of single-cell profiling of vascular tissue from the brain and lung using Smart-seq2, *Acta2* and *Myh11* were indeed detectable in venous VSMCs, but at remarkably lower levels compared to the expression of these same genes in arterial VSMCs [77]. Thus, given the relatively low expression of *Acta2* and *Myh11* in venous VSMCs as measured even with the high read depth of Smart-seq2, the limited expression of canonical SMC genes detected in this study may result from limitations of the low read depth droplet-based sequencing technology. In addition, the zero inflation of certain genes in SMCs may further represent this cellular heterogeneity in the expression of canonical SMC genes across various SMC populations [78]. Given the established diversity and plasticity in SMC phenotypes with a spectrum ranging from expression patterns associated with contractility (i.e., aorta VSMC-like) to synthetic (i.e., fibroblast-like) [79, 80], these related cell types may be difficult to differentiate without prior FACS enrichment strategies as SMC and fibroblast cell types may cluster closely together by scRNAseq.

Despite the alignment of tdT-expressing cell populations by whole mount microscopy, flow sorting, and downstream scRNAseq that correspond to the PLV-LMCs and SSV-VSMCs, we cannot completely exclude the possibility that proposed LMCs and VSMCs in the current study may represent myofibroblast contaminants from the surrounding tissue. In particular, while noted prototypical SMC genes such as *Cald1* and *Pdgfrb* were highly expressed in the proposed SMC clusters, these genes have also been shown to be expressed by certain fibroblast subsets [77, 81]. As the identification of LMC-specific genes is currently an important goal in the field of lymphatic biology, the potential caveat of myofibroblast contamination must be considered in future investigations. With this in mind, this work does provide enormous promise towards the identification of candidate gene markers that may be used to specifically target LMCs in future studies.

Based on the myofibroblast-like expression pattern of PLV-LMCs and SSV-VSMCs, it remains unclear whether these cells may be directly related to the previously established inflammatory-erosive mechanisms of synovial fibroblasts in RA. The close proximity of the synovial lymphatics to these described fibroblast populations, coupled with the identification of peri-vascular fibroblasts involved in disease pathogenesis [4], raises the question of the relationship between these cell types in inflammatory arthritis that ought to be investigated in future studies. Overall, this study provides further insight on LMC transcriptional changes that drive the well-established role of lymphatic dysfunction in inflammatory arthritis, and highlights alterations in peri-vascular immune cell populations. Future studies will focus on further understanding the direct mechanisms between these cellular changes, and the implications on arthritic progression in the afferent joint.

## Conclusions

By scRNAseq, we have identified that chronic inflammatory-erosive arthritis is associated with reduced LMCs and M2-macrophages along with increased inflammatory monocytes in joint-draining vascular tissue. Transcriptional changes in LMCs suggest a direct role in the recruitment of immune cells and pathways known to be involved in joint inflammation, which ought to be investigated in future studies towards a further understanding of the pathogenic mechanisms associated with mesenchymal stromal cells in chronic arthritis.

## Supplementary Information

**Additional file 1 **: **Supplementary Figure 1**. Identification of *Ptprc*^+^ immune and *Prrx1*^+^ mesenchymal clusters by scRNAseq of tdT^+^ sorted cells. A feature plot is shown where *Ptprc* (CD45) identified immune cell populations **(A)**, and *Prrx1* defined the mesenchymal cells containing the smooth muscle cell (SMC) populations and fibroblasts **(B)**. Low quality cell clusters that remained in the dataset were identified by relatively high mitochondrial gene expression indicating dead or dying cells, noted by the arrow **(C)**. Blue = high expression, grey = low expression. **Supplementary Figure 2**. Differentially expressed genes in cell clusters identified with integration of WT and TNF-Tg datasets. The wild-type (WT) and tumor necrosis factor transgenic (TNF-Tg) single-cell RNA-sequencing (scRNAseq) datasets were integrated together, and unsupervised shared nearest neighbor (SNN) clustering in Seurat resolved 20 distinct cell clusters. The top 3 genes (duplicates omitted) for these 20 cell populations are shown as a heatmap, and the cell populations correspond by color and cluster order. The cell numbers for each population are noted in parentheses, and depict the same populations shown in the UMAPs of Fig. 3. The full gene list defining the cell populations is provided in the Supplementary Materials. **Supplementary Figure 3.** Micro-CT confirmation of severe inflammatory-erosive arthritis in the experimental TNF-Tg mice. Ex vivo micro-computed tomography (μCT) was performed on the ankle joints of the wild-type (WT) **(A-B)** and tumor necrosis factor transgenic (TNF-Tg) **(C-D)** mice used in this study. The intact ankle in WT mice **(A)** shows the expected bone architecture with the talus segmented in blue **(B)**. In contrast, a representative image of a severely eroded ankle in the TNF-Tg mice is depicted **(C)** with the talus identified in red **(D)** as an established biomarker of arthritis. The talus bone volumes were quantified in Amira software with a significant decrease in talus volumes in TNF-Tg mice compared to WT **(E)**. An unpaired t-test was used for statistical analysis (**** p < 0.0001). **Supplementary Figure 4**. Gene expression patterns define various monocyte and macrophage cell populations. Immune cells were first identified by *Ptprc* (CD45) expression **(A)**, and myeloid cell populations were defined by *Itgam* (CD11b) enrichment **(B)**. Patrolling monocytes were defined by *Cx3cr1* **(C)**, while inflammatory monocytes were identified by *Ly6c2* **(D)** and *Ccr2* **(E)** expression. M1-polarized macrophages were also enriched in *Ccr2* and *Ccl2* **(F)**, while M2-polarized macrophages were identified by *Mrc1* **(G**, CD206**)** and *Cd163* **(H)** expression. Notably, the M2-macrophage population was also enriched for *Lyve1* **(I)** known to regulate collagen content in smooth muscle cells [38]. and M2-macrophages were selectively decreased in tumor necrosis factor transgenic (TNF-Tg) mice as noted in Fig. 3. Feature plots overlaying the UMAP are provided to visualize the change in expression (grey = low, blue = high gene expression). **Supplementary Figure 5**. Additional lymph and angiogenic genes and muscle-related transcription factors selectively expressed in the SMC clusters. Along with the genes indicated in Fig. 4, *Vegfa* (lymphatic muscle cells (LMCs) 1.10, vascular smooth muscle cells (VSMCs) 1.23 log_2_ fold-change (log2FC) in **A**), *Vegfd* (LMCs 1.47, VSMCs 1.00 log2FC in **B**), and *Angpt1* (LMCs 0.29, VSMCs 0.51 log2FC in **C**) as factors critical for lymphangiogenesis and angiogenesis were selectively expressed by LMCs and VSMCs compared to all other cell clusters. In addition, *Vegfd* known to interact with VEGFR3 to promote lymphangiogenesis [56] demonstrated significantly greater expression in the LMC population compared to VSMCs (0.47 log2FC in **B**). However, *Vegfc* expression was nearly undetectable in all cell clusters **(D)**. In addition, muscle-related transcription factors such as *Prrx1* (LMCs 2.22, VSMCs 2.42 log2FC in **E**), *Twist1* (LMCs 1.71, VSMCs 1.19 log2FC in **F**), *Tead1* (LMCs 0.64, VSMCs 0.38 log2FC in **G**), *Meox2* (LMCs 0.28, VSMCs 0.94 log2FC in **H**), *Id3* (LMCs 2.37, VSMCs 2.66 log2FC in **I**), and *Csrp2* (LMCs 1.06, VSMCs 1.91 log2FC in **J**) were significantly increased in muscle cell populations compared to all other sequenced cell clusters. While these transcription factors are not individually specific to smooth muscle cells (SMCs), each gene is known to regulate smooth muscle related gene expression and their co-expression in the same cell strongly indicates that the cluster is a muscle cell [47]. Violin plots are shown to demonstrate the effect size between SMC populations compared to all other cell clusters. Wilcoxon rank sum test was performed between the different cell types for statistical analysis with false discovery rate (FDR) < 2.87E-67 for all comparisons. Sample sizes: LMCs = 1,985 cells, VSMCs = 1,331 cells, Other = 5,563 cells. **Supplementary Figure 6**. LMC and VSMC clusters express fibroblast-like genes similar to myofibroblasts, but not pericyte genes. To confirm a lack of contamination by pericytes, canonical pericyte markers such as *Cspg4* **(A)**, *Mcam* **(B)**, and *Ngfr* **(C)** were nearly undetectable throughout the dataset. Note that cell sorting of tdT^+^ cells from *Cspg4*-Cre;Ai9-tdTomato as in Fig. 1 does not presume active *Cspg4* expression, but instead that tdTomato (tdT)^+^ cells at one time expressed *Cspg4*. On the other hand, non-specific fibroblast markers were highly enriched in the muscle cell populations, such as *Dcn* (lymphatic muscle cells (LMCs) 5.25, vascular smooth muscle cells (VSMCs) 5.48 log_2_ fold-change (log2FC) in **D**), *Fbln2* (LMCs 2.12, VSMCs 2.31 log2FC in **E**), and *Pdgfra* (LMCs 2.29, VSMCs 2.35 log2FC in **F**). The various validations of muscle cell identity in Fig. 1, 2, and 4 and Supplementary Figures 5 and 7 suggest that these peripheral muscle cell populations may cluster closely with fibroblasts by single-cell transcriptomics, yet exhibit distinct features to differentiate them from traditional fibroblasts. Violin plots are shown to demonstrate the effect size between muscle cell populations compared to all other cell clusters. Wilcoxon rank sum test was performed between the different cell types for statistical analysis with false discovery rate (FDR) < 2.23E-308 and the lower limit possible in R. Sample sizes: LMCs = 1,985 cells, VSMCs = 1,331 cells, Other = 5,563 cells. **Supplementary Figure 7**. Differentially expressed genes between LMC and VSMC clusters. The lymphatic muscle cell (LMC) and vascular smooth muscle cell (VSMC) clusters from the wild-type (WT) and tumor necrosis factor transgenic (TNF-Tg) integrated datasets in Fig. 3 were subclustered, and then re-integrated for analysis of differential gene expression between the populations with minimally supervised clustering **(A)**. The top 10 differentially expressed genes between LMCs and VSMCs are shown as a heatmap **(B)**, and the full list of gene expression is provided in the Supplementary Materials. Notable genes, such as *Cst3* (2.60 log_2_ fold-change (log2FC) in **C**), *Mgp* (2.75 log2FC in **D**), and *Bgn* (2.11 log2FC in **E**), were significantly increased in VSMCs compared to LMCs, while *Ackr3* (1.39 log2FC in **F**) was selective for LMCs relative to VSMCs. Feature plots overlaying the UMAP are provided to visualize the change in expression (grey = low, blue = high gene expression), while violin plots are shown to demonstrate the effect size between muscle cell populations. Wilcoxon rank sum test was performed between the different cell types for statistical analysis with false discovery rate (FDR) < 2.53E-227 for all comparisons. Sample sizes: LMCs = 1,850 cells, VSMCs = 1,466 cells. **Supplementary Figure 8**. Lack of *Acta2* expression in LMC and VSMC clusters. *Acta2* (alpha smooth muscle actin (αSMA)) is a well-established canonical marker for smooth muscle cells (SMCs), and immunohistochemistry for αSMA has been routinely used by us and others to study lymphatic muscle cells (LMCs) and vascular smooth muscle cells (VSMCs) in lymphatic and blood vessels in situ and ex vivo (Fig. 4 and [22, 43, 75, 82]). Transcriptomic studies of large arteries have also used *Acta2* expression as a phenotypic marker of SMC clusters in single-cell RNA-sequencing (scRNAseq) datasets [80, 83]. However, our scRNAseq data showed that *Acta2* expression is nearly undetectable in freshly harvested and sequenced popliteal lymphatic vessel (PLV)-LMCs and superficial saphenous vein (SSV)-VSMCs **(A, B)**. A feature plot overlaying the UMAP is provided to visualize the change in expression (grey = low, blue = high gene expression), while a violin plot is shown to demonstrate the negligible *Acta2* expression in all sequenced cell populations. We also generated *Acta2*-Cre;Ai9-tdTomato (tdT) **(C-D)** and *Acta2*-CreER;Ai9-tdT **(E-J)** reporter mice to evaluate endogenous *Acta2* gene promoter activity in LMCs and VSMCs in vivo. PLVs (dashed arrow) and adjacent SSVs (solid arrow) from a 3-month-old *Acta2*-Cre;Ai9-tdT mouse, and a 5-month-old *Acta2*-CreER;Ai9-tdT mouse (after tamoxifen induction, as previously described [22]) were harvested and immunostained for αSMA and visualized by whole mount immunofluorescent microscopy. High-magnification images of the regions with the highest tdT expression are shown. Remarkably, despite LMC and VSMC αSMA protein expression detected by immunostaining (green), tdT (red) in both reporter models was not ubiquitously expressed in SMCs of both PLVs and SSVs. Taken together, these findings suggest relatively low *Acta2* mRNA expression in LMCs and VSMCs of lower limb vessels in adult mice.

**Additional file 2.**

## Data Availability

The scRNAseq datasets generated in the current study are deposited on NIH Gene Expression Omnibus (GEO; accession number GSE190999), and source code will be made available upon reasonable request. All other datasets generated or analyzed during this study are either included in this published article (and its supplementary information files) or will be made available upon reasonable request.

## Abbreviations

TNF-Tg: Tumor necrosis factor transgenic
RA: Rheumatoid arthritis
PLVs: Popliteal lymphatic vessels
LMCs: Lymphatic muscle cells
scRNAseq: Single-cell RNA-sequencing
UMAP: Uniform manifold approximation and projection
VSMCs: Vascular smooth muscle cells
PRIME: Preinflammatory mesenchymal
SSV: Superficial saphenous vein
PLN: Popliteal lymph node
LECs: Lymphatic endothelial cells
NO: Nitric oxide
iNOS: Inducible nitric oxide synthase
SMCs: Smooth muscle cells
FACS: Fluorescence activated cell sorting
tdT: tdTomato
WT: Wild-type
DEGs: Differentially expressed genes
BrdU: Bromodeoxyuridine
DMEM: Dulbecco’s modified Eagle’s medium
FBS: Fetal bovine serum
DPBS: Dulbecco’s phosphate buffered saline
FCC: Flow cytometry core
GEM: Gel bead-in-emulsion
SNN: Shared nearest neighbor
Log2FC: Log_2_ fold-change
FDR: False discovery rate
IPA: Ingenuity Pathway Analysis
NBF: Normalized buffered formalin
αSMA: Alpha smooth muscle actin
MPI: Mean pixel intensity
μCT: Micro-computed tomography
HU: Hounsfield units
AU: Artificial units
