# Supplementary material for: Single-cell transcriptomics of popliteal lymphatic vessels and peripheral veins reveals altered lymphatic muscle and immune cell populations in the TNF-Tg arthritis model
Source: Arthritis Res Ther. 2022 Mar 7;24:64. doi: 10.1186/s13075-022-02730-z (PMC8900348; doi:10.1186/s13075-022-02730-z)
Supplement: Supplementary file 1 — Additional file 1 : Supplementary Figure 1. Identification of Ptprc+ immune and Prrx1+ mesenchymal clusters by scRNAseq of tdT+ sorted cells. A feature plot is shown where Ptprc (CD45) identified immune cell populations (A), and Prrx1 defined the mesenchymal cells containing the smooth muscle cell (SMC) populations and fibroblasts (B). Low quality cell clusters that remained in the dataset were identified by relatively high mitochondrial gene expression indicating dead or dying cells, noted by the arrow (C). Blue = high expression, grey = low expression. Supplementary Figure 2. Differentially expressed genes in cell clusters identified with integration of WT and TNF-Tg datasets. The wild-type (WT) and tumor necrosis factor transgenic (TNF-Tg) single-cell RNA-sequencing (scRNAseq) datasets were integrated together, and unsupervised shared nearest neighbor (SNN) clustering in Seurat resolved 20 distinct cell clusters. The top 3 genes (duplicates omitted) for these 20 cell populations are shown as a heatmap, and the cell populations correspond by color and cluster order. The cell numbers for each population are noted in parentheses, and depict the same populations shown in the UMAPs of Fig. 3. The full gene list defining the cell populations is provided in the Supplementary Materials. Supplementary Figure 3. Micro-CT confirmation of severe inflammatory-erosive arthritis in the experimental TNF-Tg mice. Ex vivo micro-computed tomography (μCT) was performed on the ankle joints of the wild-type (WT) (A-B) and tumor necrosis factor transgenic (TNF-Tg) (C-D) mice used in this study. The intact ankle in WT mice (A) shows the expected bone architecture with the talus segmented in blue (B). In contrast, a representative image of a severely eroded ankle in the TNF-Tg mice is depicted (C) with the talus identified in red (D) as an established biomarker of arthritis. The talus bone volumes were quantified in Amira software with a significant decrease in talus volumes in TNF-Tg mi [file 13075_2022_2730_MOESM1_ESM.docx]

**Supplementary Figure 1**

**
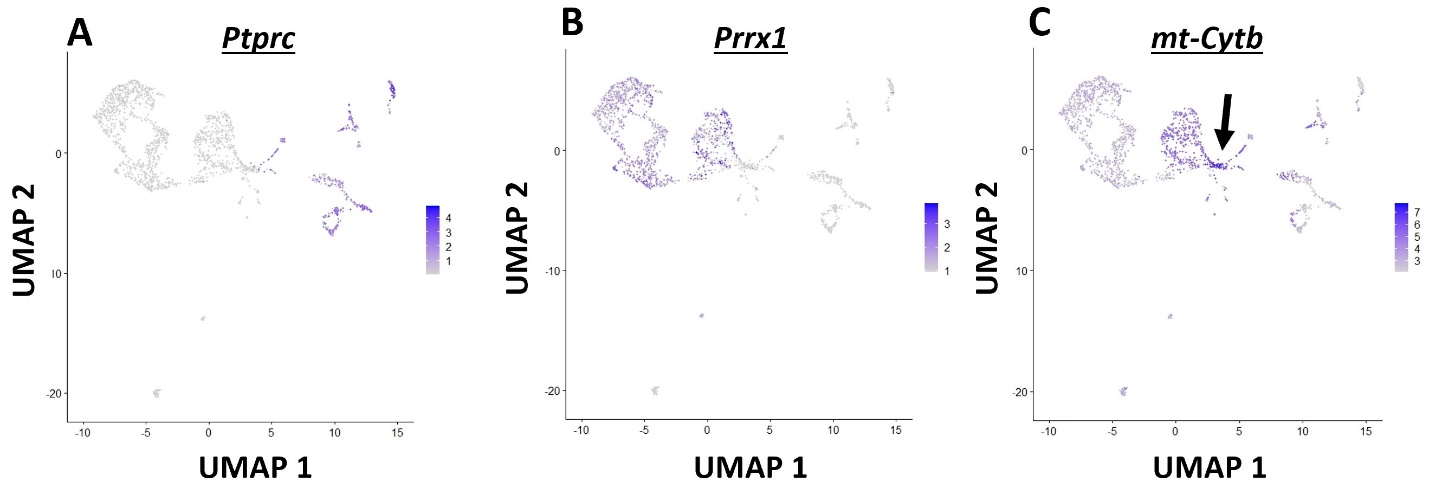
**

**Supplementary Figure 2**

**
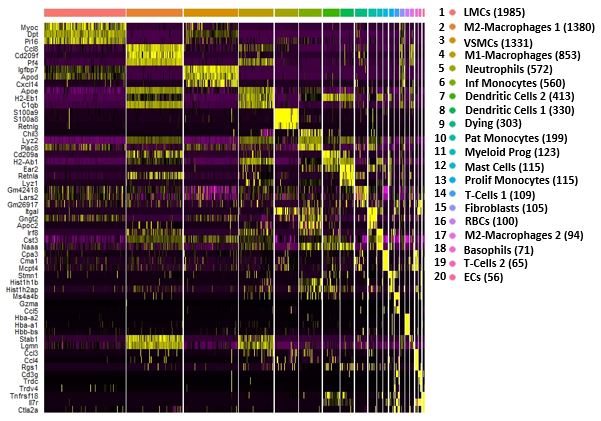
**

**Supplementary Figure 3**

**
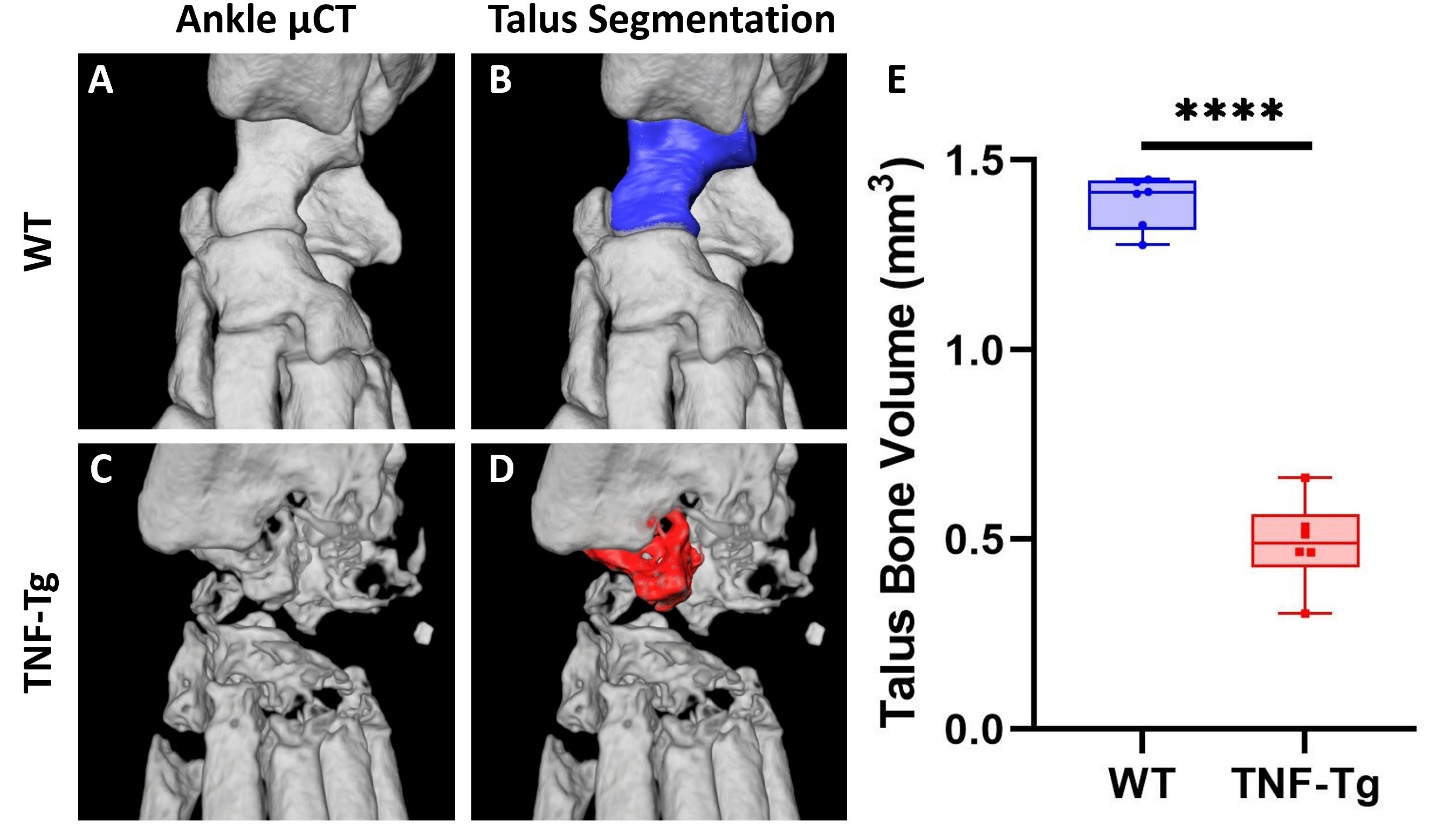
**

**Supplementary Figure 4**

**
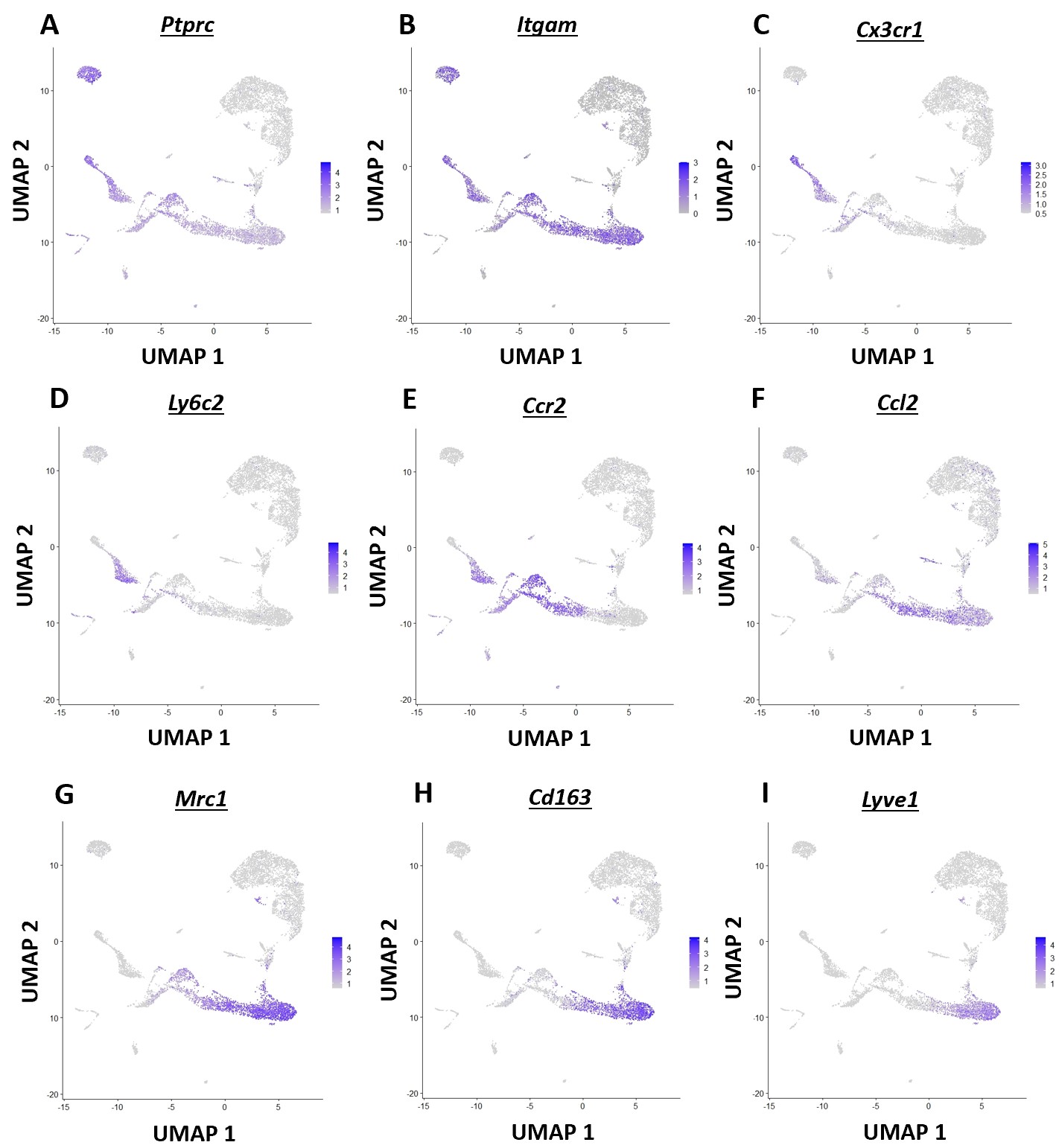
**

**Supplementary Figure 5**

**
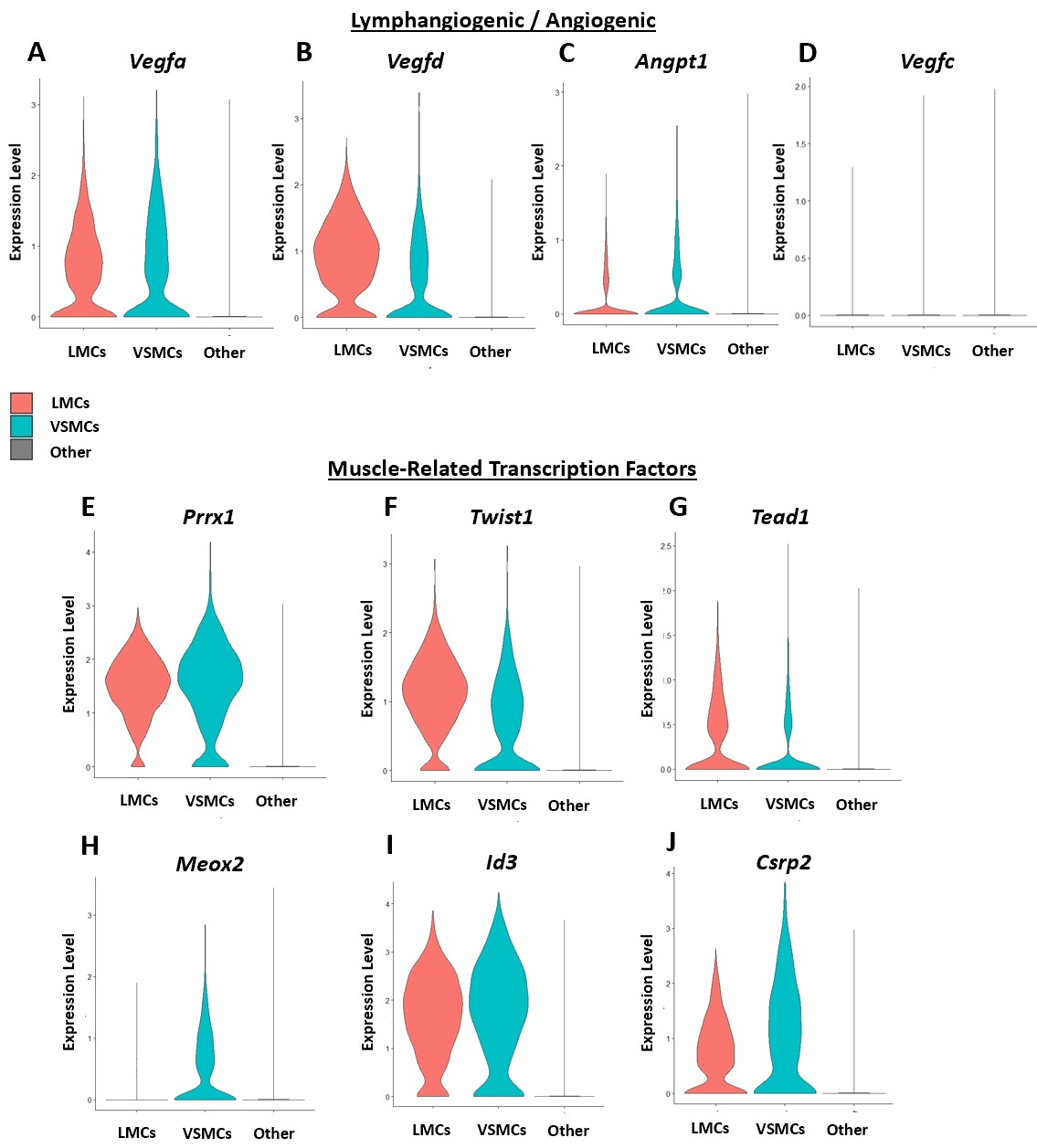
**

**Supplementary Figure 6**

**
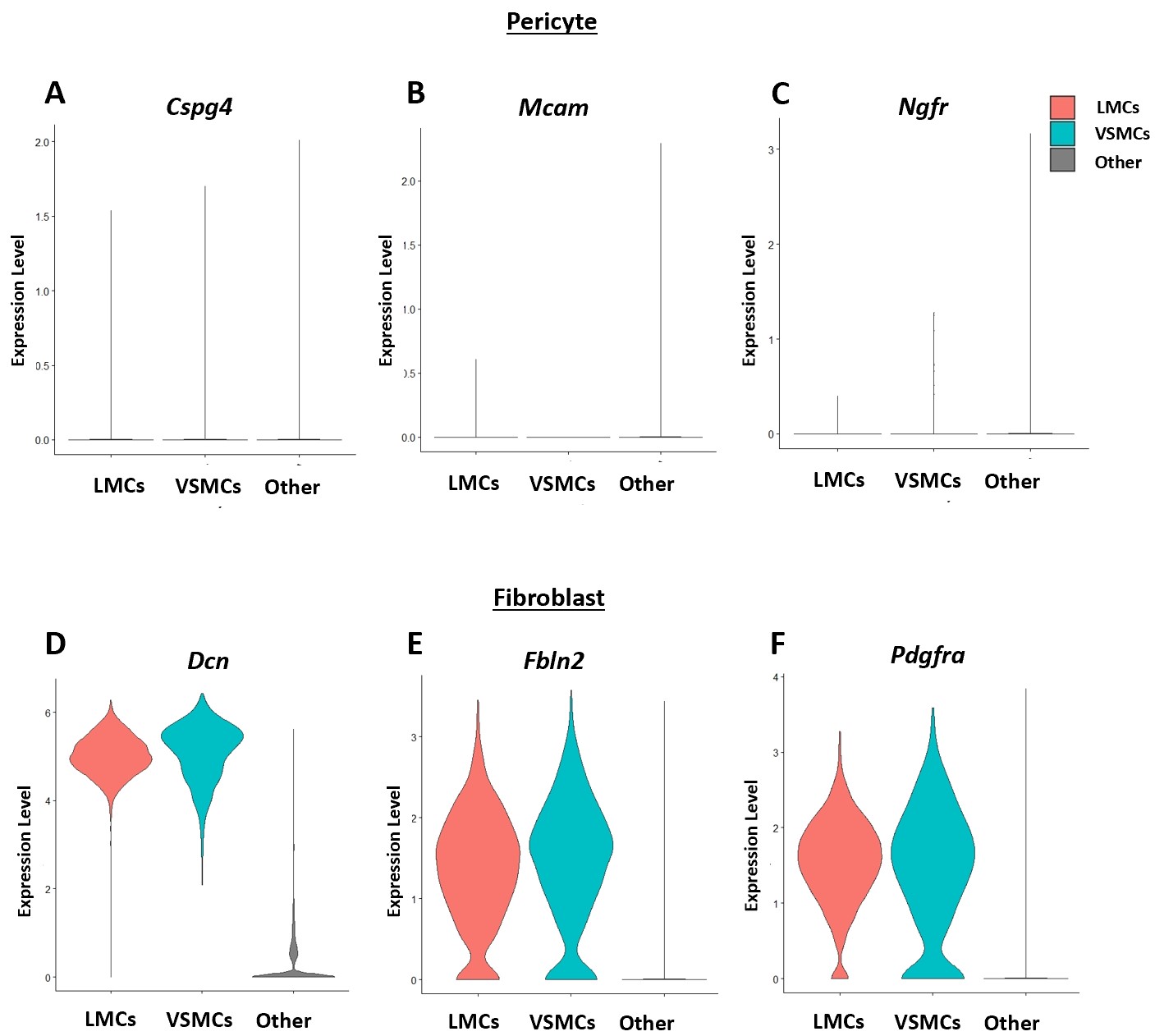
**

**Supplementary Figure 7**

**
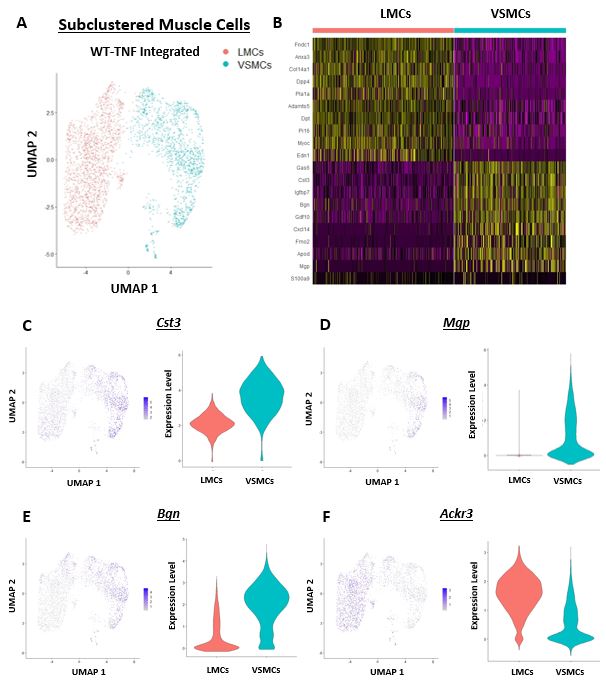
**

**Supplementary Figure 8**

**
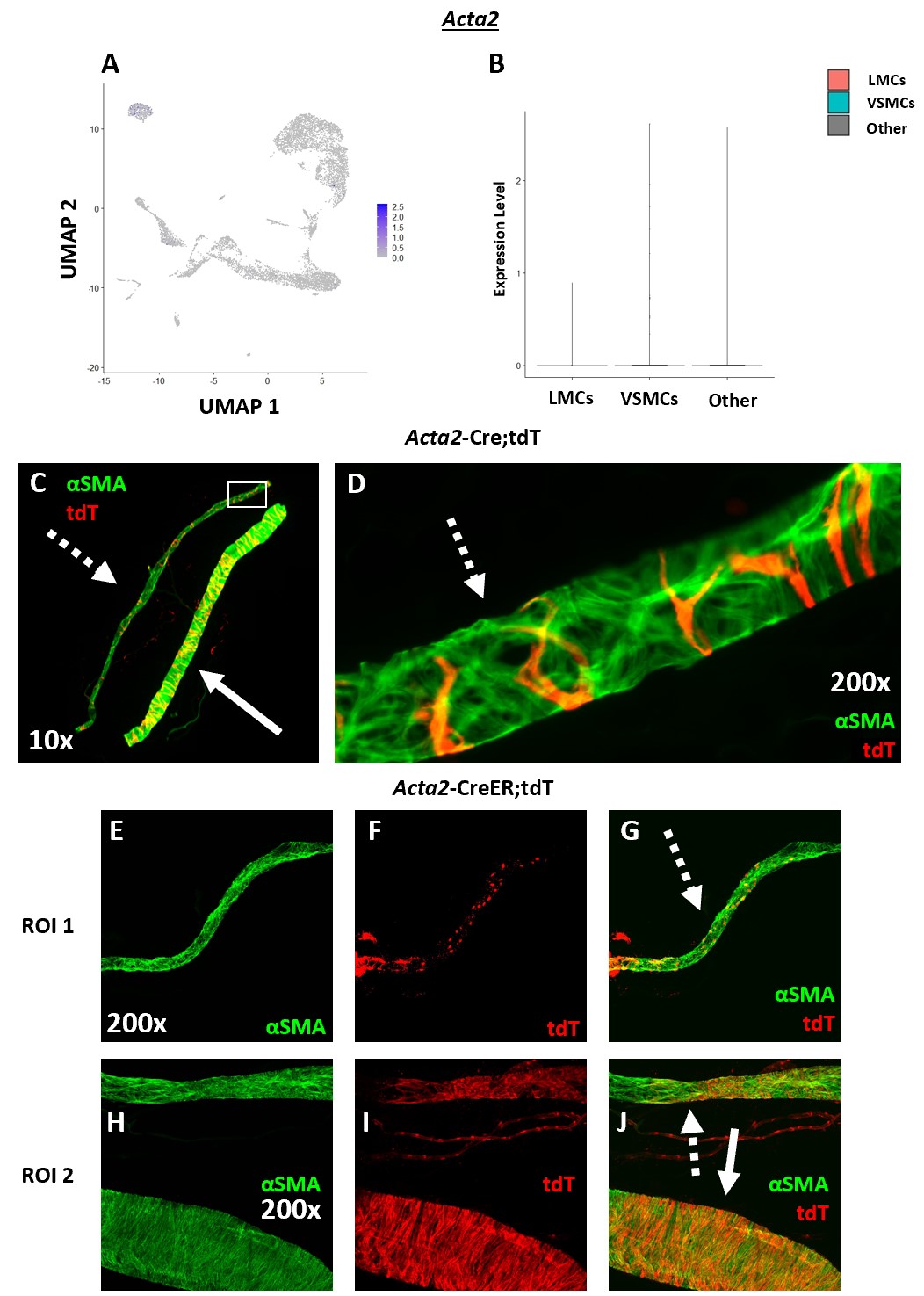
**
